# Electrospun nanofiber-based glucose sensors for glucose detection

**DOI:** 10.3389/fchem.2022.944428

**Published:** 2022-08-11

**Authors:** Yutong Du, Xinyi Zhang, Ping Liu, Deng-Guang Yu, Ruiliang Ge

**Affiliations:** ^1^ School of Materials and Chemistry, University of Shanghai for Science and Technology, Shanghai, China; ^2^ School of Health Science and Engineering, University of Shanghai for Science and Technology, Shanghai, China; ^3^ The Base of Achievement Transformation, Shidong Hospital Affiliated to University of Shanghai for Science and Technology, Shanghai, China; ^4^ Institute of Orthopaedic Basic and Clinical Transformation, University of Shanghai for Science and Technology, Shanghai, China; ^5^ Shidong Hospital, Shanghai, China; ^6^ Department of Outpatient, the Third Afiliated Hospital, Naval Medical University, Shanghai, China

**Keywords:** glucose detection, nanomaterial, enzymatic biosensor, non-enzymatic sensor, electrospun nanofibers

## Abstract

Diabetes is a chronic, systemic metabolic disease that leads to multiple complications, even death. Meanwhile, the number of people with diabetes worldwide is increasing year by year. Sensors play an important role in the development of biomedical devices. The development of efficient, stable, and inexpensive glucose sensors for the continuous monitoring of blood glucose levels has received widespread attention because they can provide reliable data for diabetes prevention and diagnosis. Electrospun nanofibers are new kinds of functional nanocomposites that show incredible capabilities for high-level biosensing. This article reviews glucose sensors based on electrospun nanofibers. The principles of the glucose sensor, the types of glucose measurement, and the glucose detection methods are briefly discussed. The principle of electrospinning and its applications and advantages in glucose sensors are then introduced. This article provides a comprehensive summary of the applications and advantages of polymers and nanomaterials in electrospun nanofiber-based glucose sensors. The relevant applications and comparisons of enzymatic and non-enzymatic nanofiber-based glucose sensors are discussed in detail. The main advantages and disadvantages of glucose sensors based on electrospun nanofibers are evaluated, and some solutions are proposed. Finally, potential commercial development and improved methods for glucose sensors based on electrospinning nanofibers are discussed.

## GRAPHICAL ABSTRACT

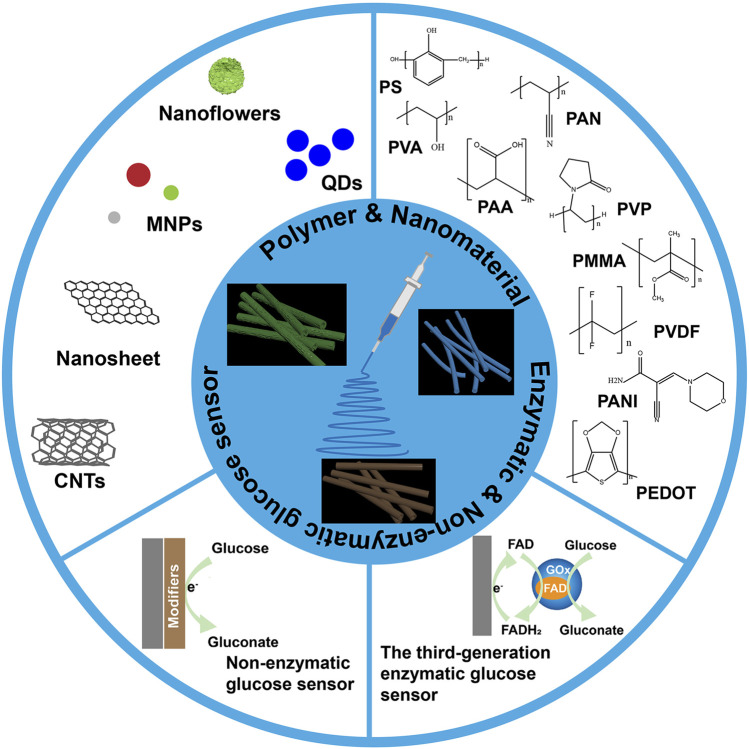

## 1 Introduction

Currently, about 537 million people have diabetes worldwide. The number of people with diabetes is increasing and is expected to increase to 783 million by 2045. Diabetes is the leading cause of death or disease in the world. If the body maintains high glucose levels, then it will continuously stimulate insulin secretion, leading to islet cell failure with insufficient insulin secretion, and eventually to diabetes (Breton et al., 2020; He et al., 2021; Hickling et al., 2021). We can distinguish between the three types of diabetes mellitus: 1) Type 1, the pancreas non-generates insulin and accounts for 10% of people with diabetes, most are young adults (Bergenstal et al., 2018); 2) Type 2, with a low rate of insulin production or the body is without insulin produced by the pancreas, accounts for 90% of people with diabetes, the majority are middle-aged or elderly (Jermendy et al., 2021); and 3) gestational diabetes, which occurs during pregnant pregnancy, and both the mother and the child have the possibility of developing diabetes (Koivusalo et al., 2016). For normal blood glucose concentrations, fasting blood glucose should be 3.9∼6.1 mmol/L and postprandial 2 h blood glucose <7.8 mmol/L. Insulin is the only substance in the body that lowers blood sugar. When glucose concentration reaches 17.0 mmol/L, insulin secretion reaches its limit. When glucose concentration drops to 2.8∼3.0 mmol/L, insulin secretion is inhibited and insulin secretion below 1.7∼2.5 mmol/L stops completely. To prevent and treat the increasing number of patients with diabetes, researchers aim to develop efficient, easy to operate, and stable monitoring devices to measure glucose levels in the stage of diabetic diagnosis.

Sensors are key to measurement equipment, and they have attracted increasing attention in the development of medical diagnosis and diabetes management (Gonzales et al., 2019). In clinical trials, blood glucose monitoring is considered to be one of the key factors in the early diagnosis of diabetes (Brown et al., 2019; Shen et al., 2021). ISO 15197:2013 is the latest self-inspection standard for blood glucose monitoring equipment and was released in 2013. In addition, the International Standards Organization (ISO) has developed a set of standards for glucose measuring equipment and blood glucose monitoring equipment, providing quality requirements and specifications to ensure that they are suitable for human use. Strict guidelines provide patients and clinicians with greater confidence in health assurance, and can help to persuade them that blood glucose monitoring devices are accurate and reliable. Home monitoring equipment can be divided into self-monitoring blood glucose (SMBG) and continuous glucose monitoring (CGM) devices. SMBG is used for self-monitoring of routine care and is used by clinicians to modify treatment with usual care. The SMBG equipment is a typical blood glucose monitor that requires a needle prick into the capillaries (Seshadri et al., 2019; Sempionatto et al., 2020; Jarosinski et al., 2021; Min et al., 2021). Yunos et al. (2021) present a radio-frequency (RF) sensor based on a stepped impedance resonator for remote blood glucose monitoring. The aim is to replace the routinely used puncture method for blood collection and instead use dipstick to measure blood glucose signals (Chien et al., 2022). Due to the high accuracy and sensitivity of laboratory CGM devices, they are used as reference techniques for calibrating other devices. Although SMBG devices are not as accurate as CGM devices, they can still provide fast and relatively accurate results for personal care, and its simpler operability compares well with the CGM devices (Sempionatto et al., 2021). The preparation of sensors without environmental influence, fast, continuous, accurate, low cost, and excellent analytical performance has received wide attention.

Electrospinning is a simple, inexpensive, and highly efficient method for the top-down production of continuous polymeric nanofibers (NFs), which is widely used in medical applications and other fields. Electrospun NFs prepared can be easily adjusted by the electrospinning processes, the size, content, and additional ingredients to change the fiber’s surface properties and structure to obtain a high level of active sites and a specific surface area (Xu H. et al., 2022). The superior sensing performance of functional nanofibers provides new approaches for the development of highly sensitive biosensors (Wei et al., 2021). NFs can directly transfer electrons from the redox active site of the enzyme to the electrode, and enhance the immobilization and electrocatalytic activity of the enzyme. Direct electron transfer on the surfaces of functional nanocomposites, which are NFs doped with conductive nanomaterials, enhances the analytical signal of the catalytic reaction on the electrode’s surface (Luo et al., 2022). The redox reaction is achieved by immobilizing the enzyme to functional nanocomposites, without requiring any mediator on the electrode’s surface (Puttananjegowda et al., 2021). This review summarizes various enzymatic and non-enzymatic glucose sensors based on polymeric nanofibers with additional ingredients. Analytical characteristics (e.g., sensitivity, detection limit, linear range, selectivity, reproducibility, stability, and response time) are an important basis for the evaluation of the glucose sensors. Meanwhile, electrospun glucose sensors are economical, and have good analytical and practical characteristics.

## 2 Background knowledge for glucose sensors

Diabetes mellitus is a body metabolic disorder (Madhavan et al., 2013). Therefore, precise monitoring and careful control of glucose levels in the blood is important for the correct diagnosis and treatment of diabetes. To understand the key of requirements for electrospinning technology to prepare glucose sensors, this review will begin by giving a brief introduction to the background of glucose sensors, including the principle of glucose sensors, sample types in glucose monitoring, and glucose detection methods in glucose sensors. According to the principles of the glucose sensors, they can be divided into enzymatic and non-enzymatic sensors (Hwang et al., 2018). Their analytical properties are improved by continuously improving the glucose oxidation mechanism in the glucose sensors. Efficient and accurate glucose detection methods are also critical for the preparation of glucose sensors. Methods of glucose detection in the NF-based glucose sensors mainly include optical and electrochemical detection (Aldea et al., 2020). CGM is an invasive measurement that requires repeated trauma to the skin to obtain samples. In contrast, non-invasive measurement detects glucose in the body’s fluids without causing harm to the body and provides blood glucose data to treat diabetes (Teymourian et al., 2020b).

### 2.1 Principles of glucose sensors

**FIGURE 1:**
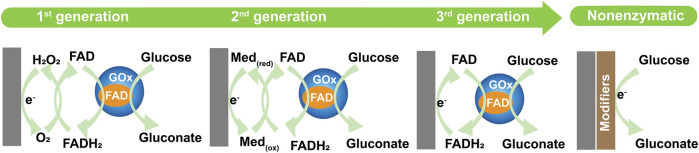
Summary of glucose oxidation mechanisms, presented as first, second, third, and non-enzymatic.

Typically, glucose sensors can be broadly divided into enzymatic and non-enzymatic glucose sensors (Zhang et al., 2017). Glucose detection involves the oxidation reaction of glucose, which generates gluconic acid (Anicet et al., 1999; Lewis and Schramm, 2003). Enzymatic glucose detection involves the oxidation reaction of glucose, reaction mediators, and enzymes. One of the most commonly used enzymes is glucose oxidase (GOx), which is used in glucose sensors as a catalyst, mainly due to the higher sensitivity and selectivity of GOx in response to glucose (Antony et al., 2019; Puttananjegowda et al., 2020a). Non-enzymatic glucose detection involves the oxidation reaction of glucose, which eliminates the need for reaction mediators and enzymes. A summary of the glucose oxidation mechanisms in glucose sensors is shown in Figure 1.

Enzymatic glucose biosensors have developed through three generations (Figure 1A). The first-generation sensor involved the oxidation reaction of glucose, reaction mediator, and enzymes, which generates gluconic acid and H_2_O_2_ by oxidizing the glucose present in the air (oxygen, O_2_), the key of reaction requires O_2_ as the reaction mediator (Naderi Asrami et al., 2020; Musse et al., 2021). The essence of glucose detection is the efficient oxidation of glucose and electron transport on the electrodes, which detects the amount of H_2_O_2_ generated, this value is then used to calculate the glucose concentration (Juska and Pemble, 2020). Because the first-generation biosensors mainly rely on the oxidation reaction of a bioactive substance, much depends on free oxygen. In addition, other electroactive species in the blood can interfere with the detection results (Zhang Y et al., 2021b). Thus, researchers developed the second-generation glucose biosensors to overcome these problems. Synthetic mediators were applied to the development of the second-generation biosensors. Most second-generation glucose biosensors use ferrocene derivatives, ferricyanide (FIC), and hydroquinone (HQ) as mediators for glucose oxidation (Teymourian et al., 2020a). The second-generation sensor involves the oxidation reaction of glucose, enzyme, and synthetic mediator. These media transport electrons from the redox active site of the enzyme to the electrode, thereby participating in glucose oxidation reactions in place of oxygen. However, the second-generation biosensors still face challenges from the distractors, mediator size, and diffusion molecules. The potential leaching of the electrode and nearby enzyme, and the instability of the response can both hinder the utility of second-generation glucose biosensors (Guven et al., 2021). The incorporation of nanomaterials not only reduces the enzyme leaching rate and ensures the sensor reproducibility but also improves the electron transfer rate so that the biosensor has a higher sensitivity (Liu et al., 2020a). The third-generation sensor uses the oxidation reaction of glucose, enzyme, and substrate for the immobilization of the enzyme (nanomaterials), and therefore the mediator is eliminated. In the third generation of glucose biosensors the enzymes are in direct contact with the probe and the electrons are transferred directly from the enzyme to the electrode, with a low working potential close to the redox potential of the enzyme itself (Sehit et al., 2020). Therefore, nanomaterials can promote electron migration between the enzyme active site and the working electrode, which results in a rapid reaction in the third-generation biosensors. Nanostructures play a key role in the third-generation biosensor electrodes, and high-surface-area of nanostructures increase the electrical active area (Naresh and Lee, 2021). The use of nanofibers as a substrate for immobilization of the enzyme show excellent results because of their high-surface-area, which allows for better action between the electrode and enzyme to increase the electron transfer rate in the glucose biosensors (Bollella et al., 2017).

For the enzymatic glucose biosensors, the most important point is the immobilization with high stability of the enzyme on the appropriate substrate (Lipińska et al., 2021a). However, these biosensors lack long-term stability because of the properties of the enzyme. Moreover, the sensing ability of these enzymes during the measurement process is very easily affected by changes in pH, temperature, and time. In addition, the price of the enzymes is relatively expensive and not suitable for mass use. As a result, the development of enzyme-independent glucose sensors, or non-enzymatic glucose sensors (considered fourth-generation sensors), has received more attention to improve the shortcomings of enzyme biosensors (Dhara and Mahapatra, 2018). The principle of the non-enzymatic glucose sensor is that it causes glucose oxidation directly on the electrode’s surface in the absence of other mediators and enzyme (Gonzales et al., 2019; Kim J et al., 2019b) (Figure 1B). Selectivity is important in the analytical characteristics of the glucose sensors, and therefore improving the anti-interference ability of these sensors is critical (Naikoo et al., 2022). Glucose sensors can suffer from interference from substances that may electrochemically react at the sensor’s working electrodes, and the generator of the reaction can give interference detection results (Dhara and Mahapatra, 2018). The biological species (e.g., dopamine and uric acid) in blood can easily be oxidized, which interferes with the electrochemical detection of glucose (Martinkova et al., 2019). They can also exhibit cross-reactivity with the enzyme, and the structurally glucose-associated molecules suffer from the possibility of cross-reactivity (e.g., fructose, lactose, mannose) (Lorenz et al., 2018). In addition, pH values and commonly used medications (e.g., ascorbic acid and acetaminophen) are also factors that interfere with the measurement results (Nguyen T. et al., 2019). In this review, an analysis of enzymatic and non-enzymatic glucose sensors is used to understand the role of electrospinning technology in glucose sensors.

### 2.2 Sample types for glucose measurement

Currently, blood glucose measurement and analysis samples mainly rely on an invasive (finger puncture) blood test. However, repeated wound puncture can cause massive bleeding, infection, and even fainting and other serious problems (Teymourian et al., 2020b). Because diabetic patients need continuous and long-term monitoring of blood glucose levels, non-invasive glucose monitoring of human body fluids has been explored by many researchers. External fluids in the NF-based glucose sensor measurement include tears, saliva, and sweat (Ates et al., 2021; Li and Wen, 2021). The most important problem of non-invasive glucose sensor is that the glucose content in external fluids is much lower than that in blood, which requires a non-invasive glucose sensor that has higher sensitivity.

**FIGURE 2:**
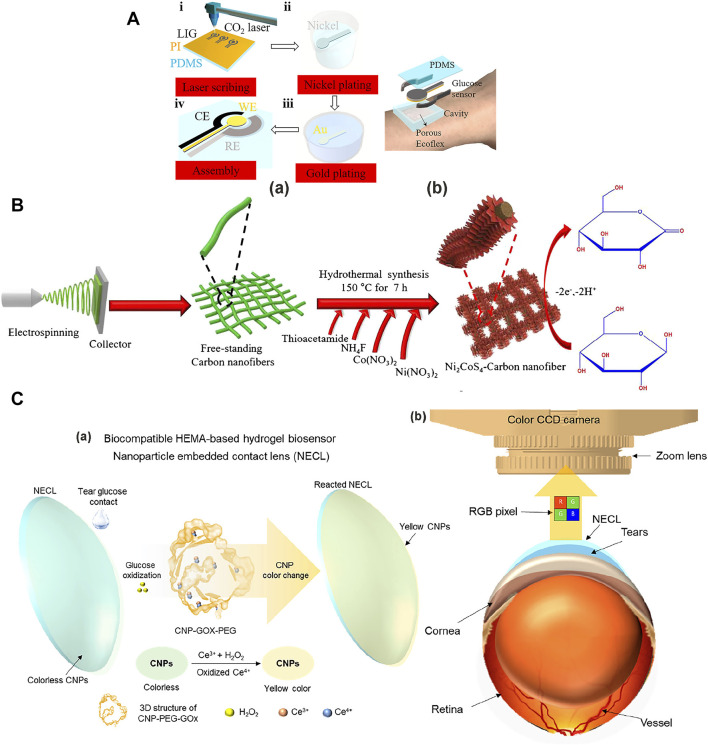
Glucose sensors used to detect glucose in sweat, saliva, and tears. **(A)** Laser-induced graphene non-enzymatic glucose sensor to detect the glucose in the sweat. **(a)** Fabrication process of the non-enzymatic glucose sensor electrodes. **(i)** Laser scribing to prepare LIG electrodes. **(ii)** Ni plating and **(iii)** Au plating. **(iv)** Fabrication of working, reference, and counter electrodes. **(b)** Schematic illustration of a wearable glucose sensor (Zhu J. et al., 2021a). **(B)** Fabrication process of the non-enzymatic Ni_2_CoS_4_-CNF/GCE electrodes to detect the glucose in the saliva (Ezhil Vilian et al., 2021). **(C)** Colorimetric NECL and optical monitoring system to detect the glucose in the tears. **(a)** The glucose oxidation turns the NECL yellow in color depending on glucose concentration. **(b)** A color CCD camera (Jeon et al., 2021).

Sweat is a very important biological fluid in non-invasive biosensing technology because sweat contains rich chemical biomarkers that are closely related to physiological health assessment and it is easily collected from the skin after it is secreted by skin pores (Li Jiankang et al., 2021). Sweat contains about 99% water, and the rest includes electrolytes, calcium, potassium ions, and metabolites [e. g., glucose (0.01–1.11 mM)] (Alahnomi et al., 2021). Sweat glucose sensor technology provides a solid platform for analyzing blood glucose levels in human subjects in real time (Katseli et al., 2021; Manjakkal et al., 2021; Tseng et al., 2021). However, sweat glucose levels are too low in healthy people. Therefore, we need to improve the sensitivity of sweat glucose sensors. Zhu et al. (2021a) made a laser-induced graphene non-enzymatic glucose sensor (Figure 2A). The 3D porous LIG foams or fibers were coved on a flexible, thin-film substrate, which was followed by electroless plating of Ni and Au. The Au/Ni/LIG electrode exhibits a high sensitivity of 3,500 µA mM^−1^ cm^−2^, with a large linear range 0–30 mM and a low detection limit of 1.5 µM. The prepared sensor has a high sensitivity and a suitable linear range to detect trace glucose in sweat.

Saliva is a kind of body fluid and is produced by salivary glands. It is also the most easily available body fluid in the human body. Therefore, saliva has also become a popularly studied glucose monitoring biological liquid (Golcez et al., 2021). Current salivary glucose levels have been shown to positively correlate with blood glucose levels (Bergenstal et al., 2018). Human salivary glucose levels are about 0.008–1.77 mM (Tseng et al., 2021). Because the level of glucose concentration in saliva is only 1%–10% of the glucose concentration level in the blood, its detection equipment needs to have extremely high sensitivity (Wei et al., 2021; Chakraborty P et al., 2021a). Ezhil Vilian et al. (2021) fabricated the Ni_2_CoS_4_ nanomaterials on carbon nanofibers by electrospinning and hydrothermal route to measure the glucose in saliva (Figure 2B). Nanostructures were grown on a doped tin oxide (FTO) coated glass substrate by chemical bath deposition. The Ni_2_CoS_4_-CNF/GCE electrode exhibited glucose sensitivity 6.201 µA nM^−1^ cm^−2^, with the linear range 5–70 nM and a low detection limit of 0.25 nM, along with fast response time by the amperometry method. The lower concentration linear range and the extremely high sensitivity all meet the detection requirements of glucose levels in saliva.

Tears are an emerging body fluid used for medical monitoring (Tam et al., 2021). The sweat and saliva blood glucose monitoring problems include the sample collection methods, sample contamination (skin dust and oral wounds), and bias caused by skin exposure different environments, oral drugs, and proteolytic enzymes (Chakraborty P et al., 2021a). Tears usually contain more than 20 different substances, including water, electrolytes, proteins, metabolites (e.g., glucose), and trace metals. The glucose levels in the tears also correlate well with the plasma glucose levels, namely the tear glucose concentration (0.05–5 mM) (Kim J et al., 2019b). Jeon et al. (2021) designed a nanoparticle embedded contact lens (NECL) for an optical monitoring system (OMS) to detect glucose in tears (Figure 2C). Cerium oxide nanoparticles (CNPs) were used as the chromogenic substrate (Ce^3+^ to Ce^4+^) to make the GOx/CNPs/PEG (poly (ethylene glycol)) NECL of OMS for tear glucose measurements. The OMS exhibited the fluorescence turn-on for the glucose detection concentration range of 0.1–0.6 mM, with a low detection limit of 0.1 mM. This shows its practical application potential for rapid field detection. Analysis of the sample protected *in vivo* ensures the continuity of monitoring and the cleanliness of the sample.

Samples and materials in invasive and non-invasive glucose sensors are listed in Table 1, with details given on their sensitivity, detection limit, and detection range. Comparing the analytical characteristics of the invasive and non-invasive glucose sensors, invasive (blood) samples have a wider glucose detection linear range and a lower sensitivity than external body-fluid samples. The narrow and low linear ranges with high sensitivity comply with the conditions of glucose detection in external body fluids. However, these techniques suffer from limitations in glucose detection. The addition of electrospun NFs allows non-invasive glucose detection combined with more techniques to improve the analytical characteristics.

**TABLE 1:** Invasive and non-invasive glucose sensors, with analytical characteristics.

Sample	Material	Linear range (mM)	LOD (µM)	Sensitivity (µA cm^−2^mM^−1^)	References
Blood	CHIT (GOx)/AuLrTiND	0.04–15.05	1.75	23.47	Lipinska et al. (2021a)
		15.05–40.00		10.63	
Sweat	Au/Ni/LIG (LIGF)	0–4 @ 0.5 V	1.5	1,200 (LIG)	Zhu et al. (2021a)
		0–30 @ 0.1 V		3,500 (LIGF)	
	3D-PMED	0–1.9	5	35.7	Cao et al. (2019)
Saliva	Porous NiO nanostructures	0.005–0.825	0.084	2,432	Chakraborty et al. (2021a)
Tears	GOx/CNPs/PEG	0.1–0.6	0.1 mM	—	Jeon et al. (2021)
	a-GQD/PBA	0–20	2.1	—	Tam et al. (2021)

### 2.3 Methods of glucose detection

Glucose detection methods include Raman scattering, fluorescence emission, luminescence quenching, colorimetric, and electrochemistry, and so on. Although electrochemical detection can perform accurate quantitative analysis, practical applications require complex equipment operations (electrochemical energy transfer systems) and sample preparation. The colorimetric glucose sensor based on visual detection can be used for qualitative analysis, with simple operation and visual observation, but has poor quantitative measurement. Given that most NF-based glucose sensors use electrochemical detection and the colorimetric detection method, this review presents only these two methods.

**FIGURE 3:**
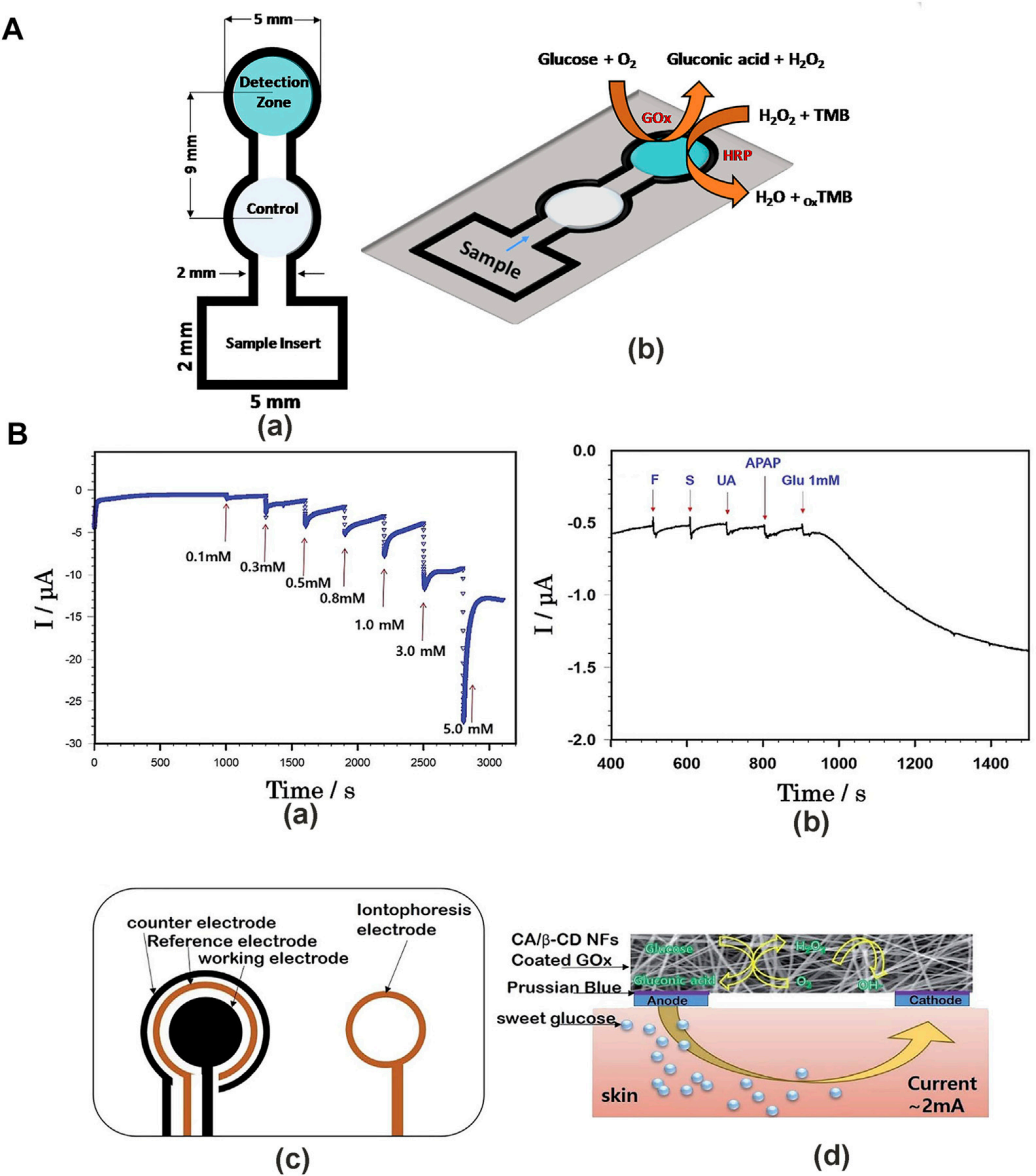
Detection methods for a glucose sensor. **(A)** The CNP-PEG-GOx colorimetric glucose sensor. **(a)** Colorimetric glucose sensor mold. **(b)** Glucose levels were measured in tears using TMB as a colorimetric substrate (Gabriel et al., 2017). **(B)** Mechanism of cellulose/β-CD/GOx NFs glucose sensor. **(a)** Amperometric responses of glucose in PBS at −0.2 V. **(b)** The effect of interfering substances on measuring glucose levels. **(c)** Schematic illustration of iontophoresis printable electrodes, counter, reference, and working electrode for glucose sensors. **(d)** Mechanism of iontophoresis on the epidermal cellulose/β-CD/GOx NFs (Kim et al., 2019).

In colorimetric sensors, the color change of the color-developing substance can be identified directly when the substance interacts with the analyte, without any other technical requirements (Senthamizhan et al., 2016). Colorimetric sensors neither display electromagnetic interference nor require contact with electrons (Chen and Wang, 2020). Colorimetric analysis of glucose can be qualitative—quantitative analysis can only estimate the approximate range when the accuracy is not high, Colorimetric sensor quantitative analysis can be conducted by a simple spectrophotometric measurement. At present, a large number of chromogenic materials have been applied in glucose sensors to achieve chromogenic reaction through glucose oxidation. For example, Gabriel et al. (2017) used 3,3′,5,5′-tetramethylbenzydine (TMB) as the chromogenic substrate to prepare the colorimetric sensor for tear glucose measurements (Figure 3A). To introduce chromogenic reaction into NFs, researchers usually use three techniques: polymer functionalization, changing the specific surface area of NFs, and nanofiber doping (Schoolaert et al., 2017). Polymer functionalization can solve the leaching or migration problems on sensors, and covalent ligation is an effective strategy of functionalized polymer substrate (Nguyen et al., 2014). The method of changing the specific surface area can be achieved by physical or chemical reactions, providing signal transduction to analyte interactions (Mudabuka et al., 2016). The method of placing functional matters in NFs to produce a colorimetric reaction is called doping. Chromogenic substrate, enzymes, and nanomaterials are doped with NFs for colorimetric sensor preparation. The main drawback of this technique is that the function simply physically packages the chromogenic substrate in a nanofiber polymer network where matter can permeate or migrate from the nanofiber structure (Geltmeyer et al., 2016; Najarzadekan and Sereshti, 2016). The colorimetric sensor is prepared into a blood glucose test kit and test strip. This simple instrument is inexpensive and its production is easy to scale.

Electrochemical sensor detection mainly uses the following electrochemical analysis methods. Cyclic voltammetry (CV) is a widely used electroanalysis method that provides information about electrochemical reaction rate in the analyte solution (Li Jiankang et al., 2021). The principle is that the electric current at a constant scan rate is recorded as the electrode potential varies over time between the two potential limits. The changes in the scanning rate produce corresponding results (Elgrishi et al., 2018; Chen et al., 2019; Vanýsek, 2019; Vilas-Boas et al., 2019; Wen et al., 2021). Amperometry is based on the Cottrell equation and defines the measurement of the current at a controlled applied potential as a function of time. Signal is related to the diffusion-controlled Faraday current generated by the charge transfer reaction of the analyte (Semenova et al., 2017; Wu et al., 2018; Pingarrón et al., 2020). Electrochemical impedance spectroscopy (EIS) is a useful tool for measuring the preparation and research of biosensor conductive materials, which provides changes in electrode surface phenomena and mass transfer resistance during electrochemical processes (Li et al., 2019; Abraham et al., 2020). The principle of impedimetry is to measure the complex impedance of the system as a function of the reactive small-amplitude sinusoidal electrode potential (Pingarrón et al., 2020). Differential pulse voltammetry (DPV) can detect low concentrations of analyte, with a higher current sensitivity than amperometry, and is used to calculate the lower limit of detection technology (Arellano et al., 2020; Liu J et al., 2020b; Yudan et al., 2019). Many researchers use electrospinning to make electrochemical glucose sensors to improve the accuracy of quantitative analysis. For example, Kim J. et al. (2019) prepared a cellulose/β-cyclodextrin nanofiber patch as a glucose sensor (Figure 3B). This detection method has high sensitivity and a wide detection range, which far exceeds the colorimetric sensors for quantitative analysis in accuracy and also provides a good detection platform for non-invasive sensors. However, the glucose detection devices calculate the glucose concentration by the electrochemical oxidation reaction of glucose. Therefore, it is necessary to eliminate the interference of other electrochemical reactions, such as electrochemical water oxidation reaction (WOR) or oxygen reduction reaction (ORR) (Mavrikis, et al., 2021; Shi et al., 2021).

$${2\text{H}}_{2}\text{O }⇌{\text{ H}}_{2}{\text{O}}_{2}{\;+\;2\text{H}}^{+}{\;+\;2\text{e}}^{-}\;\left(\text{WOR}\right)$$

where H_2_O is oxidized to H_2_O_2_ at the anode, via the water oxidation reaction.

$${\text{O}}_{2}{\;+\;2\text{H}}^{+}{\;+\;2\text{e}}^{-}\;⇌{\text{ H}}_{2}{\text{O}}_{2}\;\left(\text{ORR}\right)$$

where O_2_ is converted to H_2_O_2_ at the cathode, via the oxygen reduction reaction.

Due to the harsh conditions of electrochemical WOR or OOR, the high selectivity of glucose is easy to achieve (Zhang C. et al., 2022). However, the oxygen evolution reaction (OER) is thermodynamic favorability, making it easy to completely bypass the H_2_O_2_ production and directly generate O_2_.

$${2\text{H}}_{2}\text{O }⇌{\text{ O}}_{2}{\;+\;4\text{H}}^{+}{\;+\;4\text{e}}^{-}\;\left(\text{OER}\right)$$

where H_2_O is oxidized to molecular oxygen at the anode, via the oxygen evolution reaction.

The O_2_ generated during OER can cover the active site, thereby weakening the ability to oxidize glucose and the onset potential of the OER is close to the glucose oxidation potential (Reier et al., 2012). Thus, the most appropriate way to eliminate the interference is to achieve a large potential difference between the OER onset potential and the glucose oxidation potential. Thakur et al. (2021) developed a NiVP/Pi non-enzymatic electrochemical glucose sensor where the OER interference can be overcome by fine-tuning the metal ratio. This offers a new approach towards the electrochemical detection of glucose that eliminates the OER interference.

The development of colorimetric sensors is limited by their long response times and low sensitivity. There are still plenty of areas where colorimetric sensors can be improved, including chemical, thermal instability, and cumbersome manufacturing procedures. Electrospun NF-based colorimetric glucose sensors achieve higher sensitivity and shorter response times. Electrochemical glucose sensors use transfer electrons to achieve an electrochemical detection method. Due to its superior detection range, low detection limit, and high sensitivity, this sensor can be better used for non-invasive biosensing modes in different biological liquids. However, electrochemical detection requires the application of complex, difficult electrochemical equations, and the resulting data require secondary processing. In contrast, colorimetric detection through the naked eye can directly obtain the results, its operation is simple, and it is more popular.

## 3 Electrospun glucose sensors

### 3.1 Electrospinning technology

**FIGURE 4:**
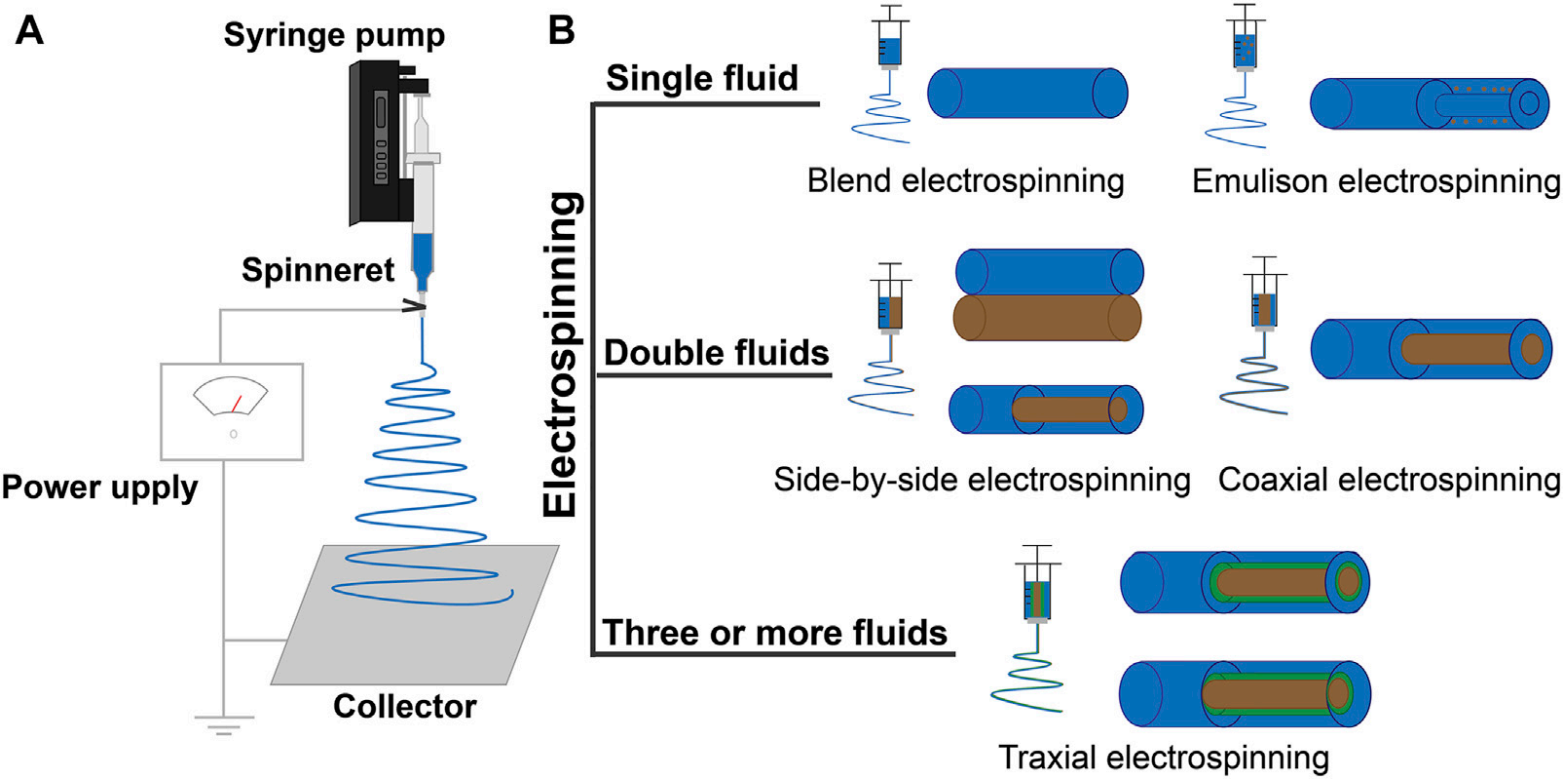
**(A)** Schematic diagram of a typical electrospinning apparatus. **(B)** Classification of electrospinning processes.

Electrospinning was initially an electrohydrodynamic method for converting the filament-forming polymers into nanofibers (Wang Y et al., 2021b; Chen, et al., 2022; Ji, et al., 2022). It has drawn increasing attention recently because active ingredients (including those for detecting glucose) can be easily encapsulated into the polymeric nanofibers to form a new kind of functional nanocomposites (Wang M. et al., 2021; Zhang M. et al., 2022; Zhou Y. et al., 2022). Based on this strategy, electrospun nanofibers have found potential applications in almost all types of scientific fields, such as energy, environment, medicine, tissue engineering, food, and agriculture (Wang Y. et al., 2022; Zhang et al., 2022d; El-Shanshory et al., 2022; Guo et al., 2022; Song et al., 2022; Yuan et al., 2022). Its popularity has a close relationship with its capability to create nanofibers, and also because this is a nano era (Yu, 2021; Yu and Lv, 2022). A typical electrospinning apparatus has four key elements: a power supply, a syringe pump, a spinneret, and a collector, as shown in the diagram of Figure 4A. The power supply often has two selections, alternating current or direct current (Sivan et al., 2022; Sriwichai and Phanichphant, 2022). The applied voltages are often between 5 and 30 kV. Syringe pumps are exploited to drive the working fluids to the spinneret in a quantitative manner (Liu et al., 2022c; Liu et al., 2022a). The spinneret is the most innovative section in an electrospinning apparatus (Wang M. et al., 2022; Liu et al., 2022b). In the literature, the spinneret is relied on to determine what kinds of electrospinning processes are used; for example, a mono-axial spinneret leads to a single-fluid blending electrospinning, a concentric spinneret is used for a coaxial electrospinning, an eccentric spinneret is used for a side-by-side electrospinning process, and spinnerets with complicated structures are used for multiple-fluid processes (Kang et al., 2022; Liu et al., 2022e; Liu et al., 2022d). Many sorts of collectors are used to randomly collect the nanofibers and aligning them into a certain order. The four sections of an electrospinning apparatus work together to prepare the nanofibers. Besides some parameters of the apparatus (such as nozzle diameter, power supply, and collector types), there are a wide variety of parameters that influence the formation of nanofibers. These parameters can be divided into three categories. The first category includes the parameters of the working fluids (e.g., polymer types, molecular weight of polymer, solvent, additives, viscosity, conductivity, and surface tension). The second category includes the operations parameters, such as the applied voltage, the flow rate of fluid, and deposition distance of nanofibers (Zhan et al., 2021). The third category includes the parameters of the working environment, mainly the relative humidity, and temperature (Guo et al., 2021; Liu et al., 2021a; Liu et al., 2021b; Zhao et al., 2021a). Although a very simple and direct one-step process for creating nanofibers, the mechanism of electrospinning is extremely complicated due to the overlapping of several disciplines (Zhou et al., 2022b; Zhou et al., 2022a). Many early investigations focused on the typical three-step working processes; that is, the Taylor cone, the straight fluid jet, and the instable bending and whipping region (Guo et al., 2021; He et al., 2021; Nauman et al., 2021). A cone shape is formed when the applied voltage is large enough to overcome the surface tension of a droplet pumped out from the nozzle of a spinneret, the famous Taylor cone. Later, a fluid jet is emitted from the top of Taylor cone, which is followed by gradually increased loops thanks to the complicated electric repulsion (Lv et al., 2022; Yousefi et al., 2019). After these three steps, the solvents in the working fluids are exhausted and solid nanofibers deposit on the collector to form a random film (Brimo et al., 2021; Feng and Hao, 2021).

### 3.2 Electrospinning processes

This section will describe the continuous progress of the electrospinning process; that is, single-fluid, double-fluid, and multiple-fluid electrospinning (Figure 4B). Single-fluid electrospinning is the first occurrence and the simplest electrospinning process, which mainly includes blend electrospinning and the emulsion electrospinning (Zhang et al., 2021a). Metal-oxide NFs made by blend electrospinning have attracted extensive attention in the research of non-enzymatic glucose sensors with simple, mild, and efficient virtues. Lu et al. (2014) prepared CuO NFs by electrospinning PVP/Cu(CH_3_COO)_2_ composite and then the CuO/Cu_2_O NFs were prepared by a hydrothermal method. The CuO/Cu_2_O NFs electrode, with a multiple oxidation states system, promoted the redox reactions between electrode and glucose, and the synergic effect enhanced reaction site activity. During blend electrospinning, the mixture fluid of the polymer and functional ingredients is ejected through a single needle to make nanofibers (Song et al., 2021). During emulsion electrospinning, the hydrophilic functional ingredients dissolve into the water and obtain a water/oil emulsion by diffusing in the oil phase containing the surfactant, then obtaining nanofibers with a core-shell structure by electrospinning. Li Jiankang et al. (2021) fabricated a novel fiber-optic glucose biosensor preparation using single-fluid electrospinning. The mixture of polyvinyl alcohol (PVOH) and GOx solution was prepared for electrospun NFs. The GOx was then encapsulated into a PVOH nanofiber and covered the surface of the Fiber Bragg Grating (FBG). It can be seen that nanofibers can ensure the activity of the enzyme and improve the electron transfer ability of glucose sensors by using polymer properties. The structural limitation of nanofibers by single-fluid electrospinning, the release, and immobilization of functional ingredients are not strong (Zhang et al., 2022c). Common electrospinning processes for double-fluid electrospinning include coaxial and side-by-side electrospinning (Chen J. et al., 2022; Ghazalian et al., 2022). Traditional coaxial electrospinning takes the spinnable solution as the sheath solution to contact with the environment, and the non-spinnable or spinnable solution as the core solution without contact with the external environment to form a distinct core-shell structure (Kang M. et al., 2020; Liu et al., 2021c). The flexibility of the sheath solution is limited by its “spinnability.” Researchers have proposed an modified coaxial electrospinning, in which the sheath solution is non-spinnable, and the core solution is non-spinnable or spinnable (Yu et al., 2010; Ning et al., 2021). The sheath solution overcame the limitation that it must be a spinnable solution, while expanding the range of the sheath solution, regulating the nanofiber diameter, and improving the quality of the fiber (Liu et al., 2022e; Liu et al., 2022f). Ramon-Marquez et al. (2017) prepared microfluidic devices by coaxial electrospinning for optical determination glucose. The sheath solution of PolymBlend and the core solution of PMMA with PdTFPP were used to make the coaxial membrane, and then the GOx was immobilized on to the membrane by physical adsorption. Most enzymatic glucose sensors require multiple enzymes to couple the enzyme reaction, so the reaction time between enzymes is an important factor to improve the sensor’s analytical characteristics, while coaxial electrospinning can generate bi-layer enzyme-containing material that can provide a typical bi-phase enzyme controlled-release profile to get enough time for the reaction. Side-by-side electrospinning obtains the Janus structure of the nanofibers, which is also one of the most basic structures and differs from the traditional core-shell structure where its two chambers are separated and parallel or eccentrically arranged together, with both solutions in contact with the surrounding environment (Xu L. et al., 2022). The eccentric spinneret creates Janus nanofibers with high quality. Multi-fluid electrospinning uses three or more different fluids through the triaxial, multiaxial electrospinning, or multi-channel electrospinning technologies to make fibers with special nanostructures (Wang et al., 2020; Zhao et al., 2021b; Lv et al., 2021; Yu et al., 2021). Later, methods for designing nanofibers with complex structures were reported, such as quad-fluid coaxial electrospinning and tri-fluid side-by-side electrospinning (a coaxial electrospinning with a side-by-side core and a coaxial electrospinning with an acentric eccentric core). The electrospinning processes and detection methods used in glucose sensors are listed in Table 2, with the polymer materials and functional ingredients. Various electrospinning processes with polymer materials and functional ingredients have been applied to glucose sensors. At present, a variety of polymer materials have been tried as scaffold materials and different properties of functional ingredients have been introduced, but the preparation of glucose sensors still mostly uses single-fluid electrospinning (Aldea et al., 2021; Li Jinze et al., 2021). Multiple-fluid electrospinning processes are less used in the development of glucose sensors, and therefore they have high application development value in the future. The significant benefits of preparing glucose sensors using electrospinning are as follows:

(1) Polymers with different characteristics can use double-fluid or multiple-fluid electrospinning processes to prepare conductive nanofibers and substrates, to load various functional ingredients, and improve the electron transfer capacity of the sensor.
(2) The high porosity of nanofibers can uniformly and effectively immobilize various functional nanomaterials on the surface of nanofibers, and promote the electrocatalytic activity of the electrodes.
(3) The high flexibility of electrospun NFs helps the sensor to easily handle stabilization and reproduction.
(4) NF-based sensors with high specific surface area are good immobilization substances for enzymes, providing more active sites for the redox reactions of glucose.
(5) NFs can achieve the high sensitivity and selective characteristics of sensors, and can be used in continuous blood glucose monitoring devices (Xu X. et al., 2022).
(6) Electrospinning is a simple and effective method with various materials for nanofibers.

**TABLE 2:** Electrospinning processes and methods of detection in glucose sensors.

Polymer material	Functional ingredient	Electrospinning process	Detection method	References
PAN	Copper acetate, cobalt acetate	Blend	Electrochemic	Ding et al. (2020)
	Nickel chloride, cobalt chloride	Blend	Electrochemic	Guo et al. (2020a)
	Ce (NO_3_)_3_·6H_2_O	Blend	Electrochemic	Kumar et al. (2021)
PVP	Co (NO_3_)_2_·6H_2_O, Fe (NO_3_)_3_·9H_2_O	Blend	Electrochemic	Ding et al. (2021)
	Fe (NO_3_)_3_·9H_2_O, Cu (NO_3_)_2_·3H_2_O	Blend	Electrochemic	Xia et al. (2018)
PCA, PAEK	Cupric acetate, Cadmium acetate	Blend	Electrochemic	Liu et al. (2019a)
PMMA	GOx, PdTFPP	Coaxial	Luminescence quenching	Ramon-Marquez et al. (2017)
PU	GOx, o-dianisidine, HRP	Coaxial	Colorimetric	Ji et al. (2014)

From different standpoints, electrospinning technology has attracted extensive attention in the application of 1D nanomaterials. Different structural nanofibers, such as porous structures (Zhang et al., 2022c), beads-on-string structures (Liu et al., 2022b), hollow structures (Liu et al., 2022c) and tri-chamber complex nanostructures (Wang et al., 2020), are prepared directly or indirectly (post-treatment) by regulating the electrospinning parameters. For example, a porous structure can be regarded as a core-shell structure with some shell holes and an empty core section that can accelerate the electron transfer along the longitude direction to increase the electron transfer rate. Interestingly, different structural electrospun nanofibers could be further optimized through decoration with other sensing materials. Electrospinning technology is powerful in preparing nanostructures, which increases the diversity of nanofiber properties. These structures have promising applications in future sensors.

## 4 Materials for nanofiber-based glucose sensors

The preparation of glucose sensors using electrospinning usually involves two non-biological materials, polymers (substrate) and functional ingredients (i.e., conductivity, biocompatibility, and catalysis) (Table 3). The use of polymers as the substrate of the nanofibers ensures the immobilization efficiency of the enzyme and also has various other functions, such as catalysis and electrical conductivity (Chansaenpak et al., 2021; Naresh and Lee, 2021). The polymers in electrospun can be divided into the following categories: natural polymer, synthetic polymer, conductive polymer, and molecular imprinted polymer (Chakraborty et al., 2021a; Kim et al., 2019a). Most of the functional ingredients in electrospun nanofiber membranes are nanomaterials, which can serve both as substrates of enzymes and reaction mediators to improve the detection signal (Chakraborty S et al., 2021; Yang et al., 2020). The nanomaterials used in electrospinning can be divided into the following categories: 0D NM, 1D NM, 2D NM, and 3D NM (Gong et al., 2019; Wongkaew et al., 2019; Guo Qi et al., 2020; Sehit and Altintas, 2020).

**TABLE 3:** Enzymatic electrospun NF-based biosensors for glucose detections, with analytical characteristics.

Detection method	Material	Linear range (mM)	LOD (µM)	Sensitivity (µA cm^−2^ mM^−1^)	Selectivity test	References
Colorimetric method	GOx–HRP/PU	0.01–20	—	—	—	Ji et al. (2014)
	GOx-HRP/CS/PVA	2.7–13.8	2.7 mM	—	—	Coşkuner Filiz et al. (2021)
	GOx@HRP@TMB@Mn_3_(PO_4_)_2_-NFs	0.25–10	0.14 mM	—	NaCl, KCl, urea	Luo et al. (2022)
Amperometry	Cellulose/β-CD/GOx	0–1	0.0935	5.08	fructose, Suc, UA, AP	Kim et al. (2019c)
	GOx/Au/PMMA/PET	Up to 1.0	0.33	3.10	AA, UA, mannose, galactose, xylose	Aldea et al. (2021)
	GOx/HNF-TiO_2_	0.002–3.17	0.8	32.6	AA, DA, UA, xylose, mannose, maltose, lactose	Guo et al. (2022)
	Au/SiCNPs-PEDOT-PSS-PVDF-ENFM/GOx	0.5–20	0.56	30.75	—	Puttananjegowda et al. (2021)
	Au/PEDOT-PSS/PVDF/GOx	0–25	2.3	5.11	—	Puttananjegowda et al. (2020b)
	PAN/DDAC-Mt	0.01–2.45	2.4	52.3	Suc, fructose, AA, CA	Apetrei and Camurlu (2020)
		2.45–15		24.7		
	PAN/MB-Mt	0.01–2	3.4	56.5		
		2–8		20.5		
	CuO/PCL@PPy/ITO	0.002–6	2	NR	AA, UA, DA	Xu et al. (2018)
	Cu-nanoflower@AuNPS-GO NFs	0.001–0.1	0.018	—	AA, saccharose, urea, NaCl, KCl, BSA	Baek et al. (2020)
	PABA/f-CNTs	0.56–2.8	67	0.40 µA mm^−2^ mM^−1^	UA, AA, DA	Sriwichai and Phanichphant (2022)
	GOx/CS/GO nanofibers	0.05–20	20	1,006.86	AA, AC, UA, metal ions (Fe^3+^, Cd^2+^, Cu^2+^)	Mehdizadeh et al. (2020)
Impedimetry	PVA/PEI/GOx	0.01–0.2	0.9	—	AA, UA	Sapountzi et al. (2017)
EIS	TMC/CNs-rGO-Au-SPE	3.3–27.7	0.1 mM	9.9 × 10^−4^ KΩ^−1^ mM^−1^	—	Ahmadi et al. (2021b)
	GOx/PPy/PPy3COOH/PAN NFs	0.020–2	0.002	—	—	Sapountzi et al. (2020)
	NFZ-GQDs@GOx	0.1–6	32	—	AA, CaCl_2_, KCl, NaCl	Mercante et al. (2021)
	NFZ-rGO@GOx		14			
CV	GOx/PVA/PAA- Cu/Ni	0–33	—	0.85 µA mM^−1^	—	Kim and Kim (2017)

### 4.1 Polymer

**FIGURE 5:**
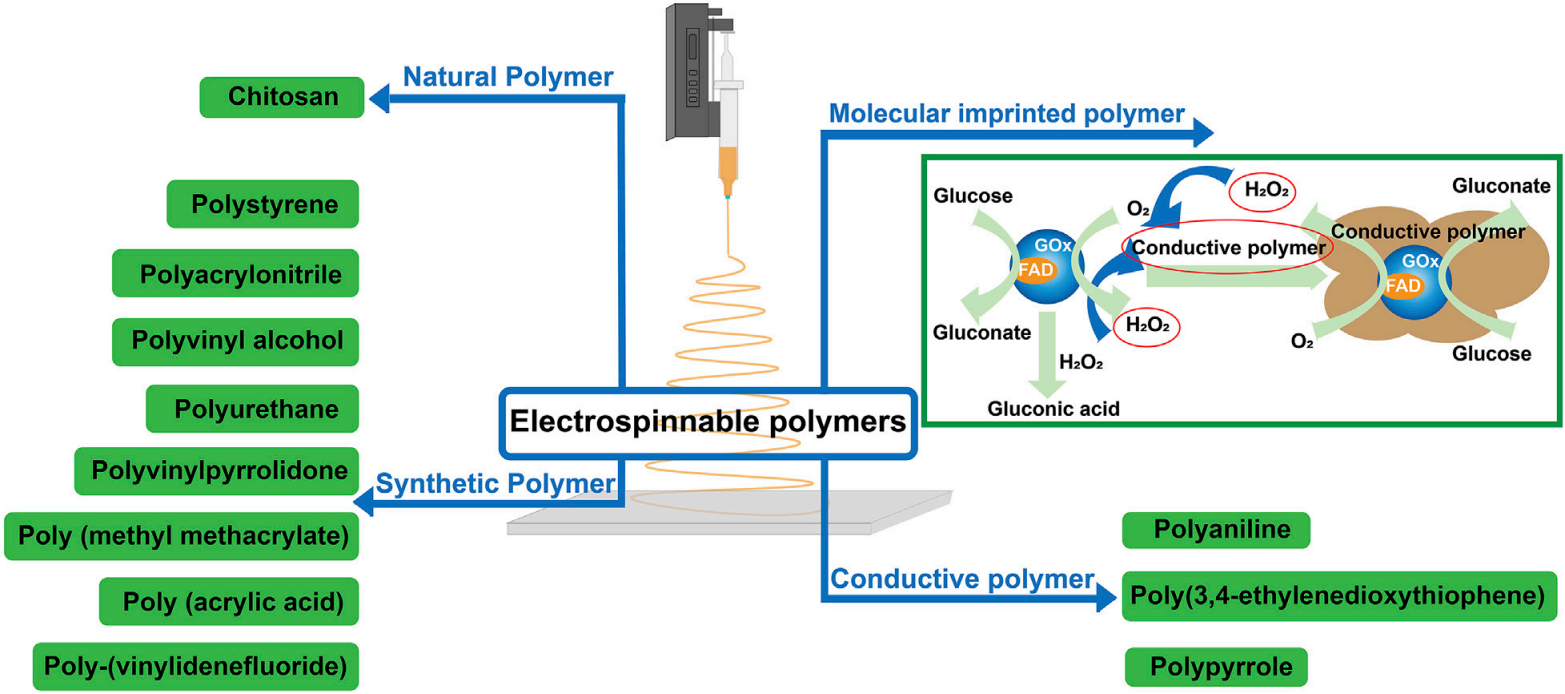
Types of polymers in electrospun glucose sensors.

Electrospun nanofibers use polymers with different functions in the preparation of glucose sensors, such as natural polymer, synthetic polymer, conductive polymer, and molecular imprinted polymer, which can ensure the immobilization efficiency of the enzyme, and have catalytic electricity and conductivity. Sensors can be prepared by making functional polymer electrospun directly into nanofibers with the induction function. The fabricating process of the glucose sensor is simple, and the nanofibers synthesized by functional polymers have a range of characteristics that can effectively improve the analytical characteristics of the glucose sensor. This review focuses on the properties and advantages of polymers in the preparation of glucose sensors by electrospinning. The types of polymers in electrospun glucose sensors are shown in Figure 5.

#### 4.1.1 Natural polymer

Natural polymer refers to the high molecular weight compounds connected by the basic structure of natural animals and plants, with good biocompatibility and biodegradability. Chitosan (CS) is a natural polysaccharide derivative deacetylated by chitin, with good biocompatibility, biodegradability, and amino activity (Kalantari et al., 2019; Bakshi et al., 2020). Aqueous solutions of organic acids such as formic acid and acetic acid are commonly used to prepare the CS spinning solution (Al-Jbour et al., 2019). Due to the difficulty of electrospinning of single CS solution, a second polymer acting as an attenuation factor enables electrospinning by limiting the hydrogen bonds between CS chains (Augustine et al., 2020). Mehdizadeh et al. (2020) made a glassy carbon electrode (GCE) that was modified with GOx/CS/graphene oxide (GO) electrospun NFs to detect glucose. The GOx/CS/GO GCE exhibited a wide linear range with a high sensitivity and low detection limit. The addition of GO and GOx to the CS nanofibers improves the conductivity and catalytic activity of the nanofibers and enables the high sensitivity of the glucose sensor.

#### 4.1.2 Synthetic polymer

Synthetic polymer is synthesized by chemical means (i.e., a single organic molecule is formed through chemical reactions with repetitive units) and because of its own uniqueness has been used the most widely in electrospinning technology.

Polystyrene (PS) is a hydrophobic polymer that uses styrene rings as a side chain and requires surface modification to introduce hydroxy groups (Lin et al., 2019). The PS is soluble in a variety of nonpolar organic solvents (such as DMF) and is one of the important materials for preparing electrospun NFs. Zhou et al. (2013) used polystyrene electrospun optical fibrous membrane (EOF) prepared by electrospinning technology, and GOx is functionalized to achieve rapid and highly sensitive optical glucose biosensors. The GOx/EOF exhibited a wide linear range with the detection limit. The good biocompatibility of PS makes it a suitable material for use in a biosensor, enabling (bt) _2_Ir (acac) to be uniformly and stably doped on the PS NFs.

Polyacrylonitrile (PAN) is a high-carbon polymer, and its carbon-carbon skeleton ensures its high biostability and degradation resistance. Nanofibers prepared by PAN have the advantages of excellent attractive properties, heat resistance, chemical resistance, and mechanical properties (Khan, 2017). Sapountzi et al. (2020) used a two-step approach to fabricate glucose biosensors. The first step uses electrospinning to prepare non-conductive PAN NFs, which are deposited on the gold electrode surfaces, and the PAN NFs are then immersed into FeTos oxidant solution. The second step uses vapor phase polymerization to prepare a conductive polymer by co-polymerizing pyrrole (Py) and pyrrole-3-carboyxylic acid (Py3COOH) on the electrode, and the Gox is immobilized by the covalent bonding method. The GOx/PPy/PPy3COOH/PAN/FeTos oxidant NFs exhibited a wide linear range with a low detection limit. The PAN was used as a scaffold material to facilitate the growth of PPy coated onto the PAN NFs surface.

Polyvinyl alcohol (PVA) is a white powdered semi-crystalline polymer. Unlike other polymers, PVA is not synthesized by polymerization of a structural monomer (vinyl alcohol). It is soluble in water—the higher the temperature, the greater the solubility—but is almost insoluble in organic solvents. While PVA has excellent biocompatibility, heat resistance, optical properties, and charge storage capabilities, the physicochemical and mechanical properties of PVA are determined by the number of hydroxyl groups present in the polymer (Aslam et al., 2018). Coşkuner Filiz et al. (2021) designed a colorimetric glucose biosensing GOx-HRP/CS/PVA nanofiber in a water-based medium by electrospinning. The naked eye colorimetric CS/PVA glucose detection strips exhibited a concentration range with a lower detection limit. Crosslinked CS-PVA NFs are suitable substrates for the immobilized GOx and HRP, improving the immobilization of enzymes and stability of colorimetric glucose sensor.

Polyurethane (PU) is a block copolymer containing low molecular weight polyester or polyether segments that are covalently attached to a polyurethane group (-NH-C(=O)-O-). Polymers are synthesized by step growth and polymerization in reactions with three basic components of isocyanate, polyol, and a low molecular weight chain amplifying agent. Ji et al. (2014) designed a new glucose test strip based on polyurethane hollow nanofiber membrane that was prepared by coaxial electrospinning. This hollow nanofiber membrane test strip can serve as either a colorimetric sensor in solution or as an optical biosensor operating in an “immersion-read” mode.

Polyvinylpyrrolidone (PVP) is a water-soluble, chemically inert, and amorphous polymer (-CH_2_CHC_4_H_6_NO-)n that is made from the monomer N-vinylpyrrolidone (NVP) through bulk polymerization and solution polymerization (Grant et al., 2021). PVP is soluble in water and most organic solvents, and has low toxicity. Huang et al. (2011) designed a biological glucose detection device by electrospun Mn_2_O_3_-Ag NFs. The PVP NFs were calcined under air atmosphere for Mn_2_O_3_-Ag NFs with the degradation of PVP. The Mn_2_O_3_-Ag/GOx platforms obtained over a glucose concentration range with a high sensitivity.

Poly (methyl methacrylate) (PMMA) is a hydrophobic amorphous polymer that is insoluble in water and soluble in organic solvents, with good chemical stability and weather resistance (Simões et al., 2019). The electrospun PMMA NFs have low mechanical properties and require increased fiber loading to enhance (Ali et al., 2015). Aldea et al. (2021) made a glucose sensor by preparing electrospun PMMA fibers, with surface-immobilization of GOx and coated with a gold layer, which was applied a high sensitivity and a low detection limit. PMMA NFs were coated on the electrode to increase the surface area and improve the stability of the glucose sensor.

Poly (acrylic acid) (PAA) is a water-soluble, anionic polyelectrolyte composed of charged carboxy macromolecules (Hamnca et al., 2022). With a large negatively charged active site, suitable for cation loading (Park et al., 2019; Ziyadi et al., 2021). The PAA has a negatively charged carboxyl group and may covalently bind to a GOx with a positively charged amine group (NH_2_) (Riccardi et al., 2017). Kim and Kim (2017) synthesized dual-functionalized electrospun PVA/PAA NFs with GOx to enhance catalytic activity. The GOx/PVA/PAA NFs were coated on Cu/Ni electrode to make the glucose sensor. The GOx/PVA/PAA NFs applied wide linear range 0–33 mM, with sensitivity 0.85 µA mM^−1^. Crosslinked PVA-PVA NFs with biocompatibility are suitable substrates for the immobilization GOx, improving the immobilization of enzymes.

Poly-(vinylidene fluoride) (PVDF) and its copolymers are one of the most challenging polymers during electrospinning (Chen et al., 2015). Poly (vinylidene difluoride)-co-hexafluoropropylene(-HFP) is a PVDF copolymer with excellent film forming capacity and rapid hydrophobicity that is widely used in electrochemical sensors due to its unique, piezoelectric, high dielectric permittivity, and thermochemical properties (Martins et al., 2014; Hartono et al., 2018; Ruan et al., 2018). Saravanan et al. (2022) made a decoration of PVDF-HFP NFs with Co-Fe metal nanoparticles serves as an electrochemical non-enzymatic glucose sensor. The of PVDF-HFP/Co–Fe membrane was applied over a wide linear range with high sensitivity, and a low limit detection and selectivity due to the high electron transfer.

#### 4.1.3 Conductive polymer

Conductive polymers have unique high conductivity, high electron affinity, strong redox activity, stability, and low cost that make it practical biosensing materials. Using conductive polymers for electrospun glucose sensors gives good conductivity, biocompatibility, and electrochemical stability, with effective immobilization of enzymes and functional materials on the nanofiber surface, which makes a direct electron transfer between enzymes and electrodes to improve sensitivity.

Polyaniline (PANI) is a π–π conjugated polymer synthesized by chemical or electrochemical oxidation of a monomer (Popov et al., 2021). The unique π–π-conjugated structure acts as a redox medium between the redox center and the enzyme electrode, facilitating electron transfer to the electrode (Neupane et al., 2021). Because PANI has strong effective reaction and anti-interference ability between enzyme and electrode, it has optimal sensing performance. The PANI is moderately conductive at low pH, but is insulated at pH 3 or 4 (Zare et al., 2020). German et al. (2020) designed a conducting polymer nanocomposite fiber glucose biosensor loaded with GOx and gold nanoparticles on the graphite rod (GR) electrode. Liu M. et al. (2019a) designed a hollow CuO/PANI NFs, electrospun PAA NFs as a sacrificial template, *in-situ* polymerization of aniline monomers to make the hollow PANI fibers and doping CuO on the PANI fibers. The CuO/PANI electrode was applied to a low detection limit, with a wide linear range. The PANI NFs have imine- and amine-groups, with high conductivity, improving adsorption ability, and reactivity of glucose sensing.

Poly (3,4-ethylenedioxythiophene) (PEDOT) is one of the most promising conductive polymers because of its excellent electrochemical activities, ion and electron transport properties, high conductivity, and stability (Abu Zahed et al., 2020). Meanwhile, PEDOT can serve as the main substrate of the biosensor due to its excellent physicochemical stability, good compatibility, reversibility, and reproducibility (Kitova et al., 2021; Liu et al., 2021d). Nashruddin et al. (2021) used PEDOT: Polystyrene Sulfonate (PSS)/Titanium Carbide (Ti_3_C_2_)/Graphene Quantum Dots (GQD) to make a label-free glucose electrochemical biosensor. The PEDOT: PSS/Ti_3_C_2_/GQD-based sensor applied a wide linear glucose range with a low limit of detection of and a high sensitivity. PEDOT:PSS has good stability, low redox potential, and poor electrocatalytic capability. PEDOT:PSS needs to be incorporated into conductive nanomaterials to stabilize electron transport and improve electrical conductivity.

Polypyrrole (PPy) is a polymer of non-toxic, highly conductivity, and porous structures, with unique molecular recognition systems and good biocompatibility, and it has been widely researched in the field of biosensors (German et al., 2021). However, because PPy cannot directly immobilize biomolecules and needs to be recombined with noble metal nanoparticles to increase the charge transfer between the enzyme and the electrode, and improve the sensitivity of the sensor. Emir et al. (2021) made an electrochemical glucose biosensor of a graphite rod electrode modified with nickel nanoparticles and PPy composite. Xu et al. (2018) designed electrospun CuO/polycaprolactone (PCL)@PPy NFs for a glucose sensor. The NFs were dipped into pyrrole solution to obtain the PCL@PPy NFs electrode and doping CuO on the electrode, which applied low detection limit, with wide linear range. The PPy NFs have high electrical conductivity, thermal stability, and great redox properties, which makes them suitable for glucose sensing.

#### 4.1.4 Molecular imprinted polymer

The molecular imprinted polymer (MIP) is an artificial recognition element that resembles a natural receptor, which specifically recognizes and binds target molecules with higher thermal and chemical stability of the MIP compared to other biometric elements (Sehit et al., 2020). By self-assembling monomer molecules at a target during formation, the fixed monomer polymerization process creates specific binding sites for the target (Asghar et al., 2019). In the MIP glucose sensor, the MIP with catalytic and conductive properties are obtained by wrapping the GOx into the conductive polymer. Crapnell et al. (2021) prepared a glucose sensor of electrospun nylon 6,6 fibers containing PPy MIPs. Added PPy MIPs in nylon 6,6 solutions were prepared as the electrospinning solutions. The PPy MIPs provided the widest linear range, with a low limit of detection. This method combines MIPs with electrospinning technology to fabricate sensing functional NFs, offering the possibility for future development of glucose biosensors using this technology.

In this review, we summarized the applications and properties of polymers in electrospun glucose sensors, and the diversity of polymer properties brings about a diversity in the performance of electrospun nanoproducts. For example, conductive materials have good electrical conductivity and natural materials have good biocompatibility. Currently, only one or two polymers are used in nanofiber glucose sensors and may be limited by the single electrospinning process (i.e., most are single-fluid electrospinning). The process of electrospinning includes single-fluid, double-fluid, and multiple-fluid electrospinning. Polymers with different characteristics can use double-fluid or multiple-fluid electrospinning processes to prepare a new functional nanocomposites. The characteristics of different polymers are used to achieve the simultaneous loading and bi-phase control of functional nanomaterials and active ingredients. Moreover, the nanofibers’ surface with special polymers or functional nanomaterials are negatively charged and biological species can also be negatively charged due to loss of protons. Under the influence of the repelling effect, the negatively charged nanofiber surfaces can strongly repel the negatively charged biological species, thus reducing the electrical oxidation of biological species on the nanofibers’ surfaces and improving the sensor’s selectivity.

### 4.2 Nanomaterials

**FIGURE 6:**
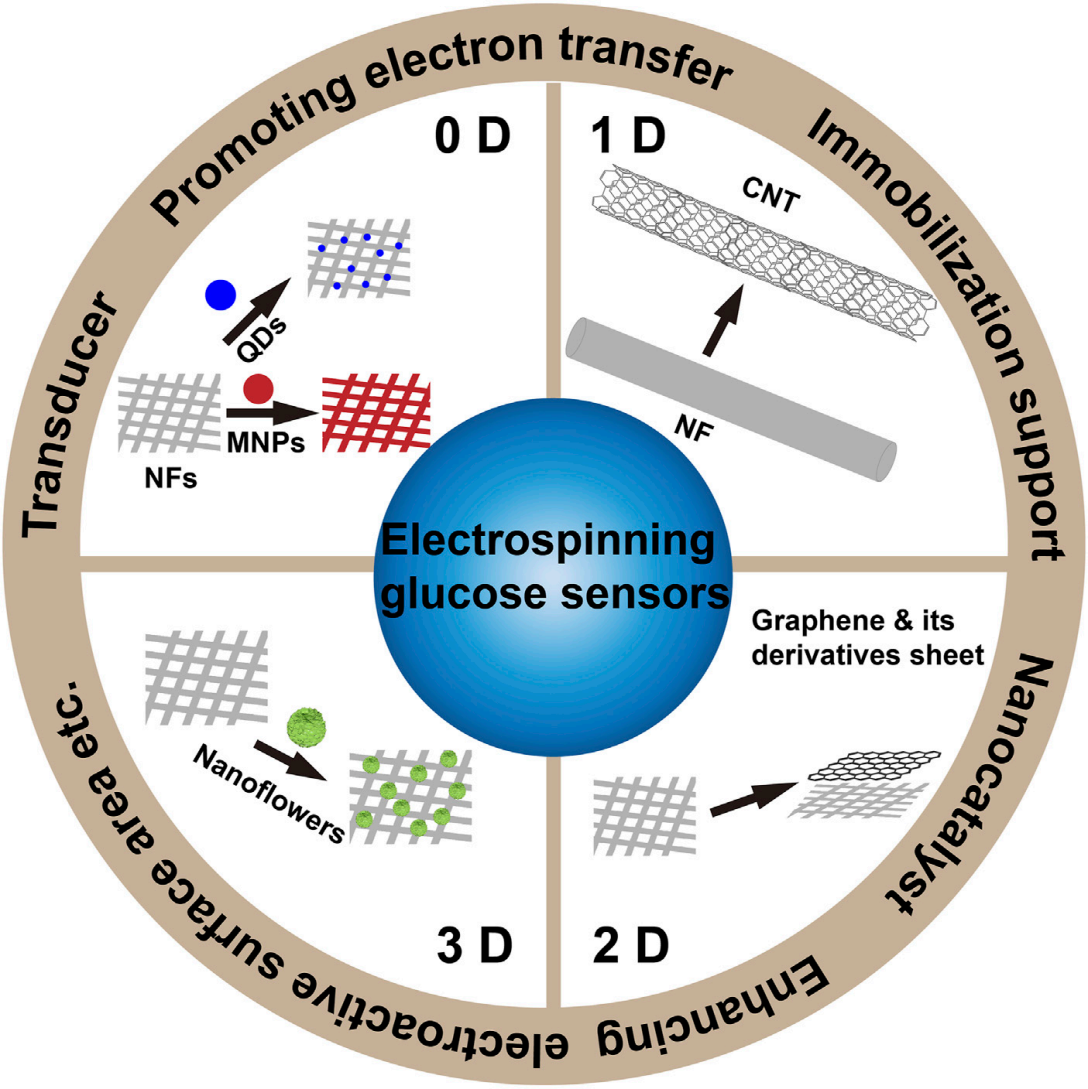
Summary of the nanomaterials in electrospun glucose sensors.

In glucose sensors, the high surface-to-volume ratio, effective enzyme immobilization, high electrical conductivity and catalytic activity are all beneficial to improve their analytical performance. The glucose sensor of NFs with functional ingredients (nanomaterials) can be fabricated by two approaches. The first is electrospun nanofibers solutions, which are fabricated by mixture of functional ingredients and polymer, followed by post-treatment, which changes the NFs’ morphology. The second is electrospun nanofibers solutions, which are fabricated by mixture of precursor and polymer, and then grown *in situ* by a catalyst on the nanofibers’ surfaces, without changing the NFs morphology. The former is simple to operate, and nanofibers with strong adsorption capacity can achieve immobilization of nanomaterials, but nanomaterials can easily gather in the polymer (Abdel-Karim et al., 2020; Guo Qi et al., 2020). The latter disperses the nanomaterials and improves the charge transfer ability, but the preparation method is difficult. Nanomaterials loaded by electrospinning can be divided into: 0DNM (NP and QD), 1DNM (CNT), 2DNM (nanosheet), and 3DNM (nanoflower) (Figure 6).

#### 4.2.1 Zero-dimensional nanomaterials

Zero-dimensional nanomaterial (0D NM) is a nanosized material in all three dimensions, including nanoparticles (NPs) and quantum dots (QDs). Metal nanoparticles such as noble metals (Au, Ag, Pt, Pd), transition metals (Fe, Cu, Co) and metal oxides (CuO, Fe_2_O_3_, MnO_2_, ZnO) can catalyze glucose oxidation, which have great advantages due to their high surface volume ratio and electrocatalytic properties (Malekzad et al., 2017; Azharuddin et al., 2019; Naikoo et al., 2021). With unique chemiluminescence and photochemical activity, QDs are useful applications in colorimetric and glucose sensing (Ramanavicius et al., 2021).

**FIGURE 7:**
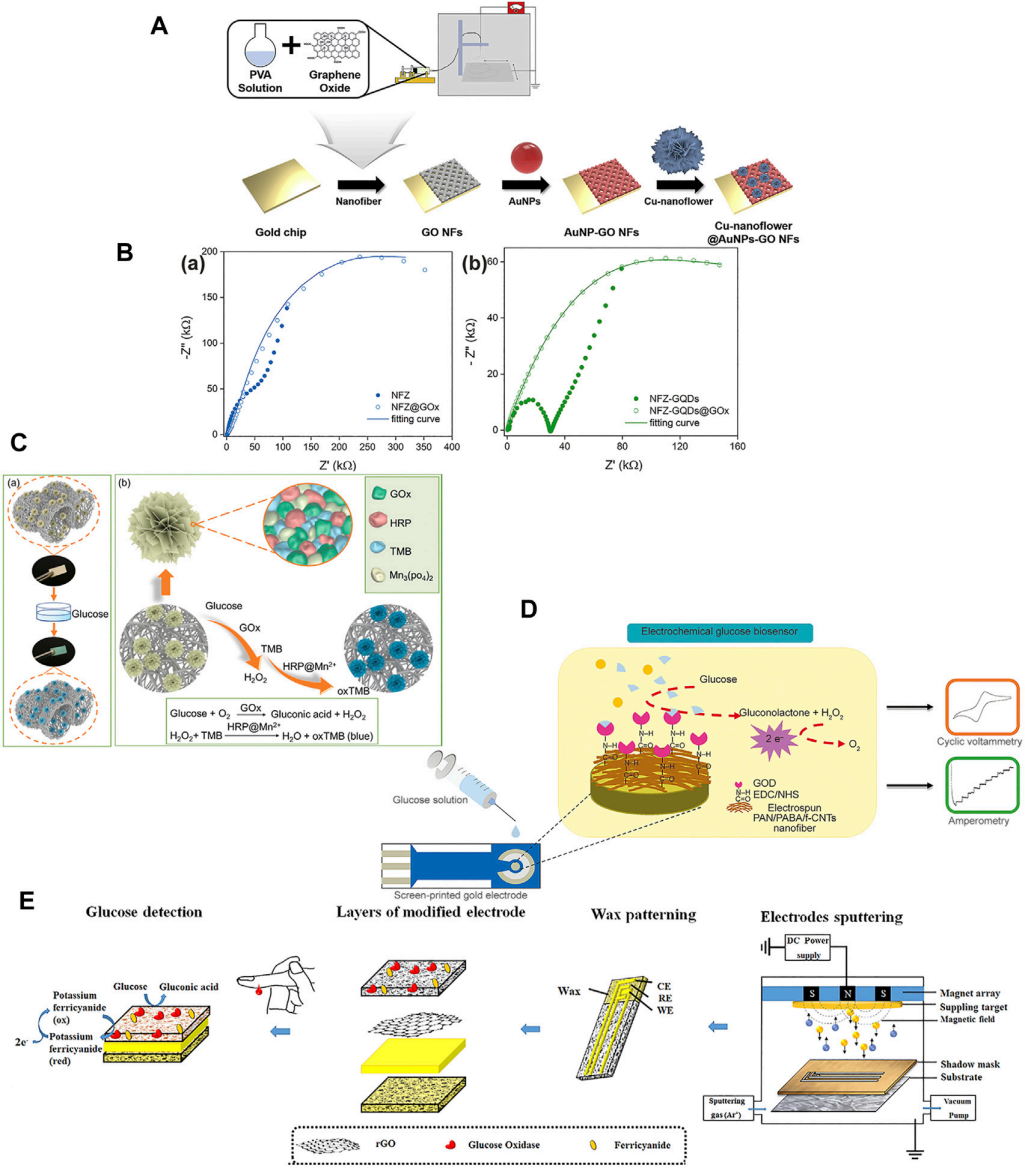
Glucose sensor loaded with nanomaterials using electrospinning technology. **(A)** Fabrication procedure of PVA/GO/AuNPs/GOx-HRP sensor, cysteamine-AuNPs solution was dropped onto the surface of PVA/GO NFs (Baek et al., 2020). **(B) (a)** Nyquist plots of NFZ and NFZ@GOx. **(b)** Nyquist plots of NFZ-GQDs and NFZ-GQDs@GOx (Mercante et al., 2021). **(C) (a)** Schematic diagram of the detection process. **(b)** Fabrication process of GOx@HRP@TMB@Mn_3_(PO_4_)^2−^ nanofibrous strip of colorimetric glucose sensor, the Mn^2+^ adsorbed onto the membrane and formed the GOx@HRP@TMB@Mn_3_(PO_4_)_2_ hybrid nanoflowers (Luo et al., 2022). **(D)** Fabrication of the PABA/f-CNTs composite film glucose biosensor based on electrospinning, the PABA/f-CNTs solution was prepared by adding f-CNTs (Sriwichai and Phanichphant, 2022). **(E)** Fabrication process of TMC/CNs/GOx-rGO-Au-SPE, SPEs were fabricated by a thick layer of Au and rGO modified the TMC/CNs substrate surface (Ahmad A. et al., 2021).

The noble metals include gold (Au), silver (Ag), platinum (Pt), and palladium (Pd). The main features of these metals are their unique biological, chemical, and physical specificities, oxidation resistance, and corrosion resistance (Li et al., 2019; Li et al., 2021c). Nanoscale noble metal materials are flexible, with good biocompatibility and catalytic properties (Basiri et al., 2018; Afzali et al., 2019; Amiripour et al., 2021; Lakhdari et al., 2021). Baek et al. (2020) designed gold nanoparticles (AuNPs), GO, and copper nanotube decorated nanofibers for use as electrochemical biosensors for glucose detection (Figure 7A). The main benefit of using AuNP is that its good catalytic properties make the glucose oxidation current higher than the other noble metals, which improves the conductivity, sensitivity, mechanical, and electrical properties of the sensor (Kumar et al., 2017; Xiao et al., 2020). The PVA/GO/AuNPs/GOx-HRP sensor exhibited a wide linear range and a low detection limit, and the high selectivity is a stronger enhancement of AuNPs nanofibers for glucose oxidation than for other biological species oxidation. Metal-oxide nanoparticles have good electrocatalytic activity and high organic trapping capacity (Kamyabi and Moharramnezhad, 2020). Nanometallic metal-oxide particles are widely used in biosensors due to their interconnected porosity, huge surface area to volume ratio, high catalytic activity, and easy synthesis properties, thus improving their sensitivity and detection limit (Saravanan et al., 2017; Liu et al., 2018; Ding et al., 2020; Ding et al., 2020; Naderi Asrami et al., 2020; Ding et al., 2021). Electrospinning is a top-down nanofabrication method that can be used to make metal-oxide nanofibers (Hsieh et al., 2016). Liu et al. (2018) designed carbon nanofibers (NiCo_2_O_4_/ECF) by using electrospinning and one-pot hydrothermal process. The NiCo_2_O_4_/ECF electrode exhibited a wide linear range, with a high sensitivity and a low detection limit. Benefiting from the synergistic effect of the properties of NFs and metal-oxide nanoparticles, the obtained electrodes have activity for direct electrocatalytic oxidation of glucose with high sensitivity (Lu et al., 2016). The metal-oxide composites with the multiple valence-states and redox-couples may boost the electron transport with high anti-interference ability (Hou et al., 2022). Metal alloys [Au-Pt, Au-Pd, Au-Ti, and Cu-Ag (Li and Du, 2017; Shim et al., 2019; Lipinska et al., 2021b; Chawla et al., 2021)] have also been used for glucose detection, and alloy electrodes can provide better electrochemical properties for electrodes to glucose cooperative activity when compared to single metal electrodes. Many transition metals have good catalytic properties, redox reaction of the transition metal centers, and can be used to prepare biosensors for application in high pH samples (Lin et al., 2018; Yang et al., 2019; Kailasa et al., 2020).

Quantum dots (QDs) are inorganic nanocrystals (NCs) that can be prepared by atoms from groups of II-VI, III I or IV-VI in the periodic table, and belong to 0D NMs (Zhou et al., 2021). They have unique photophysical properties, such as strong photostability, high brightness, and high signalability (Ramanavicius et al., 2021). Their unique chemiluminescence and photochemical activities make them attractive materials for preparing efficient biosensors to detect a wide variety of biomolecules (Yanyan et al., 2021). Biomolecular-mediated QD synthesis devices have great potential for colorimetric glucose sensing, due to the presence of different color changes during QD synthesis (Hu et al., 2020a; Ma et al., 2018). However, the underlying mechanism of the biological ligand effects of QD synthesis is unclear, which is a promising area for further exploration. Hu et al. (2020b) used a C/CdTe QDs–GOx aerogel designed microfluidic assay glucose sensor based on colorimetric detection of urine glucose that the L-Cys-mediated synthesis of fluorescent CdTe QDs. The C/CdTe QDs–GOx exhibited a wide linear range of 0–13 mM and a low detection limit of 0.223 mM. This shows that QDs glucose sensor combined with GOx has excellent selectivity, specificity, and stable for a long time. Mercante et al. (2021) designed a glucose detector utilizes zinc oxide nanofibers, graphene quantum dots (GQDs), and reduced graphene oxide (rGO) (Figure 7B). The NFZ-GQDs@GOx and NFZ-rGO@GOx platforms presented the detection range of glucose concentration was 0.1–6 mM, with a detection limit of 32 and 14 µM. According to the results, it can be found that loading QDs can distinguish between different glucose concentrations and distractors, their lower detection range and high sensitivity also provide feasibility for the applicability of non-invasive glucose sensors.

#### 4.2.2 One-dimensional nanomaterials

Carbon nanotubes (CNTs) are a one-dimensional nanomaterial (1D NM) that is composed of one, two, or several concentric graphite layers, which is topped by hollow cylindrical tubes in the Fullerian hemisphere (Sireesha et al., 2018; Karimi-Maleh et al., 2020). CNTs are the most widely studied nanotube-like material in biosensors because of their unique structure, excellent mechanical properties, high chemical stability, and high specific surface area (Pundir et al., 2021; Zou et al., 2021). CNTs have higher conductivity, like metals and semiconductors, and better electrochemical with chemical stability in both aqueous and non-aqueous solutions (Kour et al., 2020; Kumari et al., 2021). Hong-Yan et al. (2013) designed CuO electrospun composite nanofibers doped with carbon nanotubes or nickel oxide for electrochemical glucose determination. The CuO/C-NFs and CuO/NiO-NFs electrodes exhibited wide linear range, with a low detection limit and a high sensitivity. The possible synergistic effect of carbon nanofibers during catalysis to improve the detection performance. Sriwichai and Phanichphant (2022) fabricated electrochemical glucose sensor by electrospinning for poly (3-aminobenzylamine) (PABA)/functionalized multi-walled carbon nanotubes (f-CNTs) composite film (Figure 7D). The electrospun PABA/f-CNTs composite film exhibited wide linear range of 0.56–2.8 mM with a sensitivity of 0.40 µA mm^−2^ mM^−1^ and low detection limit of 0.067 mM. CNTs provide large electroactive surface area to increase electron transfer performance. In the enzymatic sensors, CNTs as molecular wires to direct transfer electrons from the enzyme active site to the electrodes.

#### 4.2.3 Two-dimensional nanomaterials

Nanosheets have an ultrathin 2-D thickness and a large area-to-thickness ratio, and most two-dimensional nanomaterial (2D NM) sensing properties depend on the surface area of the active interface (Liu et al., 2021e). The application function of the sensor can be improved by increasing the total reaction area of the nanosheets (Wang et al., 2021c). Nanosheets can be divided into inorganic nano-sheets (i.e., graphene, graphene oxide, and reduced graphene oxide nanosheets) and organic nanosheets [i.e., metal-organic framework (MOF) nanosheets] (Zhan et al., 2020). Graphene and rGO nanosheets are frequently applied in biosensors (Adeel et al., 2021; Chen et al., 2020; Jia et al., 2021; Kang S. et al., 2020; Li Jiankang et al., 2021; Lohar et al., 2021). Graphene is a carbon material with high electrical conductivity, owing to sp^2^-hybridized carbon atoms with out-of-plane π bonds (Zhang et al., 2019; Cardoso et al., 2020; Cardoso et al., 2020). Graphene derivatives, namely graphene oxide (GO) and reduced graphene oxide (rGO), usually serve as the basis for electrochemical biosensors because they optimize the electron transfer process (Huang et al., 2019; Lohar et al., 2021). GO is better water soluble and has superior electrocatalytic activity than graphene (Phetsang et al., 2021a; Wu et al., 2021). RGO nanocomposites can improve GOx activity to have a positive impact in biosensor analysis signals (Zhu et al., 2021b; Mao et al., 2021). The presence of rGO facilitates the transfer of electrons from the GOx redox center to the electrode and increases the current response of the biosensor (Phetsang et al., 2021b; Popov et al., 2021). Ahmad A. et al. (2021) designed electrochemical paper-based analytical devices (ePADs) for biosensing of glucose (Figure 7E). The cellulose nanofibers (CNs) were prepared by electrospun cellulose acetate (CA) nanofibers and deacetylating to regeneration, and were then modified with trimethyl chitosan (TMC). The screen printed three electrodes (SPEs) were fabricated by a thick layer of Au and rGO modified the TMC/CNs substrate surface. The TMC/CNs/GOx-rGO-Au-SPE presented the detection linear range of 3.3–27.7 mM, with the sensitivity increased to 9.9 × 10^−4^ KΩ^−1^ mM^−1^ and the detection limit decreased to 0.1 mM. The rGO modified the electrode to enhance electron transfer and short the response time.

#### 4.2.4 Three-dimensional nanomaterials

Nanoflowers are nanomaterials with moderate layer spacing and ultra-high specific surface area, the synergistic action of nanoalloys can promote more surface-active electron transfer during catalysis (Cao et al., 2021). At the same time, the nanoflower’s surface component is closely related to the peroxidase activity, and can improve the peroxidase simulation activity (Ma et al., 2021). Luo et al. (2022) prepared a nanofiber base band with a multienzyme-inorganic hybrid nanoflower structure for visual colorimetric detection for highly sensitive glucose detection (Figure 7C). A composite containing GOx, HRP, 3,3′,5,5′-tetra-methylbenzidine (TMB) and Mn_3_ (PO_4_)_2_ was fixed to a layered porous PVA-co-PE nanofiber band. The strips exhibited concentration range of 0.25–10 mM with a low detection limit. Therefore, nanoflowers can enhance electrochemical detection stability by promoting surface electron transfer and generate substances for colorimetric reactions by enhancing the activity of peroxidase. The small detection range and detection limit of the nanoflowers glucose sensor also provide hope for non-invasive humoral glucose detection.

Superior nanomaterials can directly improve the results of glucose detection and analysis, and can greatly improve the accuracy, specificity of the detection limit, and detection range. The response time can also be reduced by overcoming the diffusion limit. However, the same material provides different properties at different sizes, and the nanomaterials doped in the nanofibers are mostly smaller sizes. Nanofibers doped with nanomaterials can remain stable in any environmental and experimental conditions, good biocompatibility, have low toxicity during operation, reduce costs, and optimize analysis procedures. The repelling effect of the nanofiber surfaces and biological species reduces the electrical oxidation of biological species and improves the sensor good selectivity. Another factor in the improved selectivity is the stronger enhancement of nanofibers for glucose oxidation than for other biological species oxidation. In addition to these nanomaterials, there are many other nanomaterials that can be applied in glucose sensors prepared by electrospinning technology, which provide more ideas for the future development of glucose sensors.

## 5 Electrospun nanofiber-based enzymatic and non-enzymatic glucose sensors

Glucose sensors can be divided into enzymatic and non-enzymatic glucose sensing. Enzymatic glucose detection involves the oxidation reaction of glucose and enzymes, while non-enzymatic glucose sensors are based on directly electrocatalytic glucose oxidation on the electrode’s surface. Enzymatic glucose sensors have low stability, high sensitivity, and selectivity, and are affected by the characteristics of the enzyme. Non-enzymatic glucose sensors eliminate the enzyme and have high stability, low sensitivity, and selectivity. This section will review glucose sensors prepared by electrospinning to solve the problems of enzyme immobilization, and improve sensor stability and reproducibility. Functionalized nanofibers improve glucose and reduce electrochemical oxidation in other biological species, and improve the sensitivity and selectivity of non-enzymatic sensors.

### 5.1 Enzymatic glucose sensors

Enzymes can catalyze many complex metabolisms in the human body through chemical reactions, and GOx is mostly used in glucose sensors (Naghdi et al., 2018; Semwal and Gupta, 2018; Kahoush et al., 2019; Naseer et al., 2020). Glucose oxidase (β-D-glucose: oxygen-1-oxidoreductase) (GOD or GOx) is a homodimeric enzyme that is composed of two identical subunits and two non-covalently bound flavin adenine dinucleotides (FADs) (Pazur and Kleppe, 1964; Kriechbaum et al., 1989). The FAD is a very versatile organic cofactor, consisting of two main components: an adenine nucleotide and a flavin mononucleotide linked together by a phosphate group (Walsh and Wencewicz, 2012). Each GOx monomer has two distinct domains: one non-covalent but tightly bound FAD, and another bound D-glucose (Delfino et al., 2017). The GOx can oxidize glucose to D-glucose-1,5-lactone, two protons and two electrons, and the cofactor FAD forms FADH_2_. A medium where FADH_2_ can be oxidized back to FAD, leads to GOx recovery on the surface of the electrode. This process is called deep bioelectrocatalysis of GOx (Haouz et al., 1998). These processes can be displayed as follows:

$$\text{ GOx }\left(\mathrm{FAD}\right)\;+\;\beta -\text{D}-\text{glucose }\to \text{ GOx }\left(\mathrm{FAD}H_{2}\right)\;+\text{ D}-\text{glucono}-\text{1, 5}-\text{lactone}$$

Two-electron and two-proton redox reaction of the enzyme cofactor FAD is as follows:

$$\mathrm{FAD + 2}H^{+}\mathrm{+2}e^{-}↔\mathrm{FAD}H_{2}$$

The conversion of FAD to FADH_2_ in this reaction does not alter the number of β-helices in the protein shell.

The oxygen oxidizes FADH_2_ back to FAD and H_2_O_2_ is formed:

$$\text{ GOx }\left(\mathrm{FAD}H_{2}\right)+O_{2}\;\to \text{ GOx }\left(\mathrm{FAD}\right)+H_{2}O_{2}$$

$$\text{ GOx }\left(\mathrm{FAD}H_{2}\right)\;\to \text{ GOx }\left(\mathrm{FAD}\right){\text{ + 2H}}^{+}{\text{ + 2e}}^{-}$$

and this reaction regenerates GOx back to its original state.

An enzyme-catalyzed pathway during glucose oxidation is as follows:

$$\mathrm{\beta -}\text{D-glucose + }O_{2}\;\to \;\text{D-gluconic acid + }H_{2}O_{2}$$

The reaction of hydrogen peroxide is as follows:

$$H_{2}O_{2}\;\to 2H^{+}+O_{2}{\text{ + 2e}}^{-}$$

The analytical characteristics (sensitivity, detection limit, linear range, selectivity) and detection methods are listed in Table 3, with materials in enzymatic glucose sensors. Adding active ingredients in the colorimetric glucose sensors, which are GOx and horseradish peroxidase (HRP), couples the enzyme reaction for the chromogenic reaction. Glucose oxidizes with GOx, and generates gluconolactone and H_2_O_2_, and a chromogenic substrate oxidation with HRP in the presence of H_2_O_2_. Due to the nature of enzymes, enzymatic sensors have high sensitivity and selectivity but they are easily changed during the fabrication and measurement process in different pH and temperatures. When glucose biosensors are exposed to environments with unsuitable pH or temperature, the electrospinning technique can encapsulate enzymes into nanofibers to avoid inactivation. In addition, nanofibers can be fabricated with special structures by electrospinning, which helps to protect the activation of enzymes and limit the leaching of enzymes in the electrolyte solution, while increasing the GOx recovery and thus improving the reproducibility of the enzyme sensor. The general enzyme glucose sensor takes a long time to transfer electrons from the redox active site of the enzyme to the electrode surface. The functionalized nanofibers can also provide direct electron transfer to short the response time because of the steric separation between the electrode and the FAD of GOx (Guo et al., 2021; Liu et al., 2020a; Liu et al., 2020c; Nauman et al., 2021).

### 5.2 Enzyme immobilization

**FIGURE 8:**
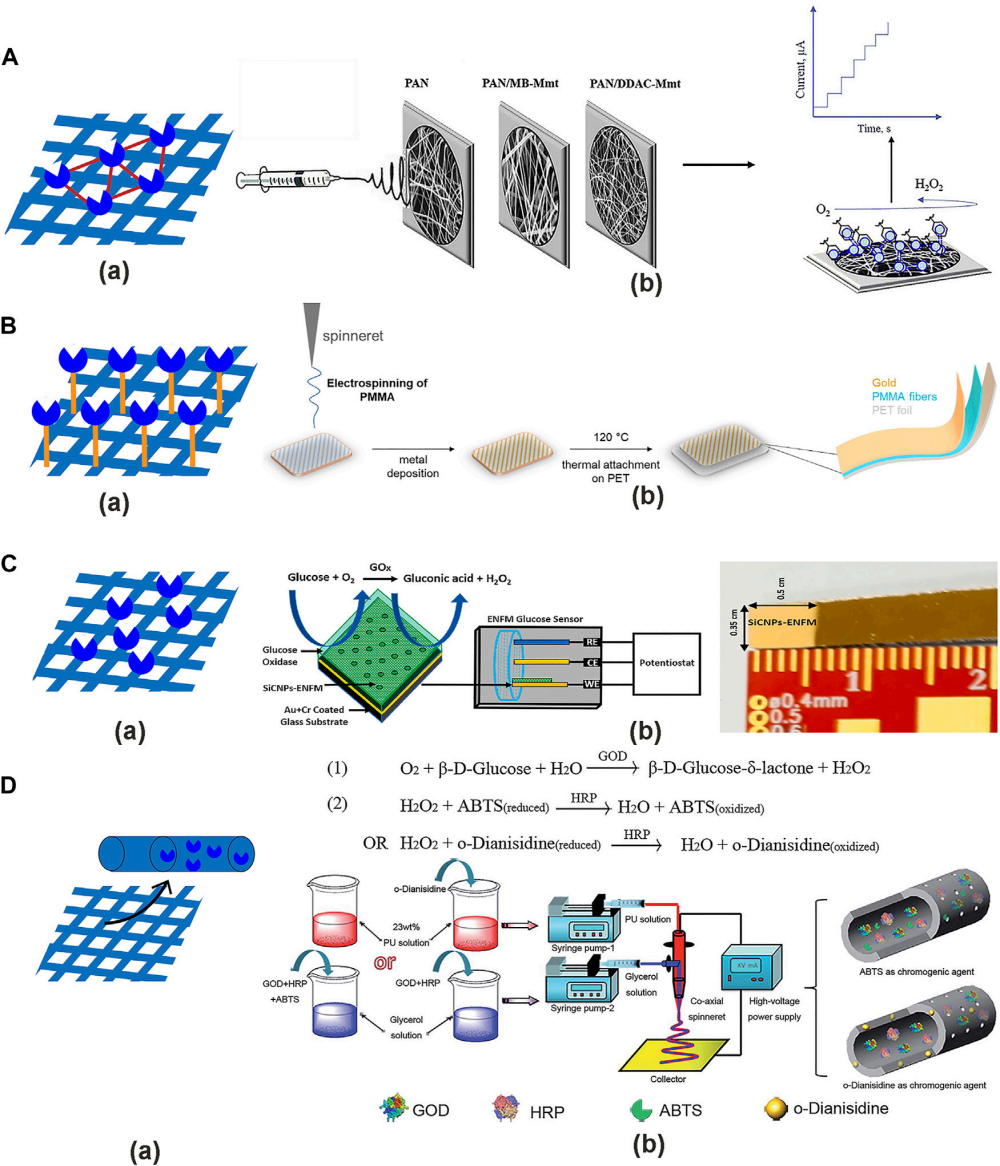
The enzyme immobilization methods on the NFs. **(A) (a)** Cross-linking method. **(b)** Fabrication of electrospun PAN/Mt NFs with MB-Mt or DDAC-Mt and immobilized the GOx by cross-linking method (Apetrei and Camurlu, 2020). **(B) (a)** Covalent bonding method. **(b)** Fabrication of Au/PMMA/PET electrode by covalent bonding method (Aldea et al., 2021). **(C) (a)** Adsorption method. **(b)** SiCNPs-ENFM glucose-sensing electrode immobilized with GOx by adsorption method and photo of the ENFM sensor electrode (Puttananjegowda et al., 2021). **(D) (a)** Entrapping method. **(b)** Glucose test strips were prepared by coaxial electrospinning and dual enzymes by entrapping method, with the glucose oxidation and chromogenic reaction (Ji et al., 2014).

The enzymes in the NF-based glucose sensors are immobilized on the NFs. The high sensitivity, selectivity, and catalytic efficiency of the enzyme gives it a high application efficiency in biosensor manufacturing, but the application of enzyme is still limited by its instability and reproducibility (Nguyen H. H. et al., 2019; Aldea et al., 2021; Ramanavicius and Ramanavicius, 2021). NFs can solve these problems because surface modification of NFs to achieve multi-point attachment can limit the undesirable conformational changes of the enzyme protein in an unfriendly environment and insoluble substrates can make soluble enzymes easier to recycle (Chen and Wang, 2020). There are four immobilization methods for the enzyme (Figure 8).

The first is the crosslinking method, where a multifunctional cross-linking reagent (glutaraldehyde) and biometric element (enzyme) are used to generate a network structure to achieve the effect of immobilization (Naresh and Lee, 2021). The cross-linking reagent forms bridges between fibers, which improves the water resistance and tensile of fiber membrane without changing the fiber morphology. The mixture is prepared by mixing enzyme solution with glutaraldehyde and dripping onto the fiber’s surface, which are then dried at room temperature. Apetrei and Camurlu (2020) fabricated electrospun PAN NFs of glucose sensors, with MB-Mt or DDAC-Mt. Electrospinning on the surface of the Pt disk electrode and then GOx was immobilized by cross-linking (Figure 8A). The advantage is that the chemical method to make the enzyme can bind more tightly to the substrate and retain the catalytic activity of the enzyme. The disadvantages are that the preparation method is troublesome and the chemical reaction distorts the partial structure of the enzyme, leading to the loss of the enzyme activity.

The second is the covalent bonding method (du Toit and Di Lorenzo, 2014). The reaction group (carboxylic group) on the polymer surface in the fiber interacts with the group of the enzyme (the side chain of the amino acid) to form a covalent bond. The formation of self-assembled monolayer is prepared by immersing the fibers in the 11-mercaptoundodecanoic acid (MUA) solution in ethanol, and the carboxylic groups of MUA are activated in a mixture of N-(3-dimethylaminopropyl)-N′-ethylcarbodiimide hydrochloride (EDC) and N-hydroxysulfosuccinimide sodium salt (NHS). Aldea et al. (2021) made the Au/PMMA/PET electrodes by electrospinning, and then immersed the electrode in a 11-mercaptoundodecanoic acid solution for immobilizing enzyme by covalent bonding method (Figure 8B). Its advantages are that the covalent bond stability and less affected by the environment. The disadvantages are that the reaction conditions are relatively strict, not easy to form covalent bonds, and the strong bond formation of enzyme and substrate is not easy to reuse.

The third is the adsorption method (Deng et al., 2020). The interaction of the enzyme with the fiber’s surface (i.e., ion, van der Waals, and hydrogen bonds) is used to immobilize the enzyme on the nanofiber surface. The immobilization of the enzyme on the fibers can be achieved by immersed or dropped the GOx solution, and dried in air, with wash in deionized water. Puttananjegowda et al. (2021) made PEDOT: PSS-SiCNPs NFs by electrospinning, and then dropped the GOx onto the nanofibrous membrane coated electrodes for immobilizing enzyme by adsorption method (Figure 8C). The advantages are that the simple process and the molecular structure of the enzyme is not destroyed to maintain a high activity. The disadvantage is that the interaction between enzyme and fiber is not very strong. The enzyme is easily shed and susceptible to environmental influence with poor stability.

The fourth is the entrapping method (Ji et al., 2014). Enzymes are immobilized in polymer semipermeable membrane substrates by electrospinning, which would be promising candidates for the development of enzymatic glucose sensors. Electrospinning technology has less effect on enzyme activity and can directly mix the enzyme into the polymer solution to electrospin nanofibers. Ji et al. (2014) prepared electrospinning solution mixing GOx and HRP in ultra-pure water. The entrapping method can be used to realize immobilization of the enzyme in the electrospun hollow nanofibers (Figure 8D). The advantage is that the high concentration of enzyme under mild conditions can effectively prevent enzyme shedding. The disadvantage is the pH value and temperature of the polymer solution are strictly controlled (Ji et al., 2017; Antony et al., 2019; Boselli et al., 2021; Coşkuner Filiz et al., 2021).

The enzyme immobilization methods and materials are listed in Table 4, together with the solvents. The scaffold materials have good stability and biocompatibility. The functional materials have excellent electrical conductivity or are suitable for enzyme immobilization. The solvents are highly volatile, and most solvents are compatible with the enzyme. In the crosslinking method, materials are suitable for the immobilization of enzymes, and enzyme activity reduction make the detection range less than the physical adsorption method. The materials of physical adsorption method need good conductivity to improve the electron transport rate. Free GOx molecules are highly susceptible to inactivation in the electrolyte solution, so a desirable immobilization method is essential to reduce the enzyme leaching rate and ensure the sensor’s reproducibility, which can achieve the full potential of the glucose biosensor.

**TABLE 4:** Enzyme immobilization methods and materials in enzymatic electrospun NF-based biosensors.

Scaffold material	Functional material	Enzyme	Solvent	Immobilization method	References
PAN	Mt	GOx	DMF	Crosslink	Apetrei and Camurlu (2020)
	—	GOx	DMF	Crosslink	Sapountzi et al. (2020)
PVA	CS	GOx	DI water, CH_3_COOH	Crosslink	
PVOH	—	GOx	PBS	Crosslink	Li et al. (2021c)
PMMA	Au	GOx	DMF	Covalent	Aldea et al. (2021)
PVP	Zinc nitrate, GQD	GOx	DMF	Physical adsorption	
PEDOT	PSS, PVDF, SiCNPs	GOx	DI water, THF	Physical adsorption	Puttananjegowda et al. (2021)
PVA	PAA	GOx	DI water	Physical adsorption	Kim and kim (2017)
PU	—	GOx, HRP	DMAc, Glycerol	Entrapment	Ji et al. (2014)

### 5.3 Non-enzymatic glucose sensors

The most common and serious problem with enzymatic sensors is enzyme instability. Enzymatic glucose sensor in thermal or chemical change, which can occur in temperatures above 40°C and pH below pH 2 or above pH 8, can cause the enzyme to inactivate (Hu et al., 2021). High or low humidity and oxygen concentrations also severely damage the sensor and affect the sensor’s signal measure deviation in the normal oxygen range (Tromans et al., 2019). A non-enzymatic glucose sensor can solve the problem of temperature, pH, humidity, and oxygen concentrations affecting the sensing. Non-enzymatic glucose detection involves the direct electrocatalytic oxidation of glucose on the electrode’s surface, which eliminates the reaction mediators and enzymes. Most electrocatalytic processes in non-enzymatic glucose sensors occur through chemisorption, in which glucose is adsorbed to the active site of the electrode. The chemisorption-based electrocatalytic process on the electrode’s surface generates reactive hydroxide species (OHads) (Burke, 1994). The consistency of the onset potential of the redox reaction with the formation potential of OHads confirms the effect of reactive OHads on the redox reaction, in which hydroxide radicals directly oxidize D-glucose (Mondal and Sharma, 2016). The electrooxidation of the adsorbates (D-glucose) is D-glucose, which is quickly oxidized into D-glucono-δ-lactone, and a further reaction into D-gluconic acid. These processes can be displayed as follows:

D-glucose →D-glucono  –  δ -lactone (Slow oxidation)

D-glucono  –  δ-lactone → D-gluconic acid (Fast hydrolysis)

The analytical characteristics (sensitivity, detection limit, linear range, selectivity, response time, stability) and detection methods are listed in Table 5, together with the materials used in enzymatic glucose sensors. Colorimetric detection method is not suitable for a non-enzymatic glucose sensor, so the glucose sensing characteristics are analyzed by electrochemical detection, mainly the amperometry. In the research, the materials in the non-enzymatic glucose sensor all contain metals because metal plays an important role in the redox reaction of glucose in the non-enzymatic glucose sensor. A non-enzymatic glucose sensor has a low detection limit and an extremely fast response time, but has a narrow detection linear range and poor sensitivity. Generally, a non-enzymatic glucose sensor eliminates the enzyme and interference of electroactive species influences the selectivity, but is unaffected by enzyme activity, with good stability and reproducibility, and can be stored for a long time. Electrospinning can easily produce nanofiber doped metals, using post-processing to fix different nanomaterials or change the fiber structure, and improve the electrocatalytic oxidation of glucose on the electrode surface with improved sensitivity. The smooth surfaces of nanofibers, with less adsorption of the intermediates during the oxidation of glucose, generate the enlarged detection range. Functional nanofibers can also effectively avoid interference from electroactive species in the electrolyte solution and improve the selectivity of glucose sensors.

**TABLE 5:** Non-enzymatic electrospun NF-based sensors for glucose detections, with analytical characteristics.

Detection method	Materials^a^	Linear range (mM)	LOD (µM)	Sensitivity (µA cm^−2^·mM^−1^)	Response time (s)	Selectivity test^b^	Number of days	References
Amperometry	PVDF-HFP/Co-Fe	0.001–8	0.65	375.01	<5	AP, DA, U, AA, K^+^, UA, Na^+^, CA	—	Saravanan et al. (2022)
	Ni/CNFs	0.002–5	0.57	—	<2	DA, AA, UA	60	Adabi and adabi (2021)
	IrO_2_ NF-Nafion/GCE	0–16	2.9	22.22	<0.5	DA, 4-AP, AA, UA	—	Dong et al. (2018)
	CuO–CdO NFs/GCEs	Up to 10	0.0527	18.76	<2	AA, UA ethanol	—	Liu et al. (2019a)
	CuO/PANI	0.001–19.899	0.45	—	—	AA, UA, DA	10	Liu et al. (2019b)
	CuO/PCL@PPy/ITO	0.002–6	2	—	—	AA, UA, DA	25	Xu et al. (2018)
	Ni (OH)_2_/ECF	0.005–13.05	0.1	1,342.2	<3	AA, DA, UA, xylose, mannose, lactose, Mal	—	Chen et al. (2017)
	Ni_2_CoS_4_-CNF-GCE	5–70 nM	0.25 nM	6.201 µA nM^−1^ cm^−2^	<3	—	—	Ezhil Vilian et al. (2021)
	Ni_2_CoS_4_/ECNF	2–10	0.93 nM	536.5	—	AA, UA, DA	5 weeks	Mohammadpour-Haratbar et al. (2021)
	NiCo_2_O_4_/ECF	0.005–19.175	1.5	1947.2	<3	DA, UA, AA, Mal, lactose, xylose, mannose	—	Liu et al. (2018)
	NiCo_2_S_4_/EGF	0.0005–3.571	0.167	7,431.96	<5	U, KCl, AA, DA, UA, lactose, xylose, mannose	2 weeks	Sun et al. (2020)
	Cu-Ni/NF	0.001–0.6	2	11.34 mA mM^−1^ cm^−2^	<2	UA, DA, AA, xylose, lactose, Suc, fructose	28	(H. Wei et al., 2021)
	CuFe_2_O_4_ nanotubes	0.02–5.5	0.22	1,239	—	AA, UA, DA	15	Xia et al. (2018)
DPV	PTBA/CuCo_2_O_4_–CNFs	0.01–0.5, 0.5–1.5	0.15	2,932, 708	—	AA, UA, DA, l-lysine, NaCl	16	Ding et al. (2020)
CV	AuNP incorporated Gr NF	0.5–9	55	1.1437	<1.7	AA, NaCl, ethanol, methanol	—	Shamsabadi et al. (2020)
	PmAPNFs/AgNPs/GCE	0.1–8	0.062	17.45	>7 s	AA, UA, DA, FA, LA, SU	—	Ahmad et al. (2021a)
	CuO NFs/GCE	0.1–10.85	0.2	483.10	—	Mal, fructose, Suc, DA, UA, lactose, AA, cholesterol	5 weeks	Khan et al. (2021)

a β-CD, β-cyclodextrin; LOD, detection limit.

b AA, ascorbic acid; UA, uric acid; DA, dopamine; CA, citric acid; Trp, tryptophan; Gly, glycine; Gal, galactose; Suc, sucrose; Mal, maltose; U, urea; AP, acetaminophen; AC, acetaminophen; NaCl, sodium chloride.

Compared with Tables 3, 5, the enzymatic glucose sensors have wider detection ranges, and higher sensitivity and selectivity than non-enzymatic glucose sensors, and are suitable for colorimetric detection. The non-enzymatic electrospun NF-based glucose sensor, retain the low detection linear range and detection limit, improve the sensitivity and selectivity, and are suitable for non-invasive detection. The gathering of high concentration nanoparticles in electrospinning solution reduces the catalytic activity and reproducibility of the glucose sensor. Thus, structurally stable nanofibers uniformly disperse the nanoparticles onto the nanofibers by covalent bonding or adsorption. Enzymes in single-fluid electrospun NFs are leached by environment influences, and the coaxial (triaxial) nanofiber composite structure reduces leaching and improves stability.

## 6 Conclusion and future prospective

In this review, it has been shown that a NF-based glucose sensor can be conveniently and functionally prepared by electrospinning for diabetic diagnosis. Electrospinning technology is a simple and general effective method to manufacture nanofibers, which greatly reduces the production cost and optimizes the production procedure. The high specific surface area and the special structure of NFs that are produced by electrospinning processes provide more active sites for the redox reaction of glucose and promote the electrochemical oxidation reaction of glucose on the electrode, which reduce the electrical oxidation of other biological species, enhance glucose oxidation, and eliminate other electrochemical reaction interference to improve the sensor’s sensitivity and selectivity. NFs are fabricated by electrospinning conductive polymers to achieve direct electron transfer between reactants and electrodes, reduce response time, and improve sensitivity. Functional NFs loaded with nanomaterials can be fabricated by *in-situ* growth and mixture solution, improving the electron transfer capability, which expands the detection linear range and improves the sensitivity of sensors. The good biological activity, high porosity, and specific surface area of the nanofibers achieve efficient enzyme immobilization of the nanofiber surface, improving stability, reproducibility, and reducing the detection limit. Methods for further modification of more stretchable nanofibers by doping different nanomaterials may provide development directions for applications in wearable devices.

Currently, the many different types of glucose sensors have advantages and disadvantages. However, future activities need *in-depth* research to solve the specific problems and should be studied on different biological fluids, including biocompatibility and real sample analysis. Most colorimetric sensors use coupled enzymatic reactions for chromogenic reactions, which requires the active site to be enhanced and strengthens the coupled enzyme reaction. The fabrication of electrospun colorimetric sensor is a one-step process, the chromogenic substrate be oxidized by single enzyme and catalytic nanomaterials, which makes the future development of a glucose detection strip feasible. Most polymers have poor electrical conductivity and need to be collected by a conductive substrate, which limits their application in electrochemical sensing and increases manufacturing costs. The electrochemical glucose sensor by electrospinning molecular imprinted polymer, conductive polymers, and conductive nanomaterials can be collected on a non-conductive substrate, which allows these devices to be developed commercially.
